# Multigram Synthesis and *in Vivo* Efficacy Studies of a Novel Multitarget Anti-Alzheimer’s Compound

**DOI:** 10.3390/molecules20034492

**Published:** 2015-03-10

**Authors:** Irene Sola, Elisabet Viayna, Tània Gómez, Carles Galdeano, Matteo Cassina, Pelayo Camps, Margherita Romeo, Luisa Diomede, Mario Salmona, Pilar Franco, Mireille Schaeffer, Diego Colantuono, David Robin, Daniela Brunner, Nicole Taub, Birgit Hutter-Paier, Diego Muñoz-Torrero

**Affiliations:** 1 Laboratori de Química Farmacèutica (Unitat Associada al CSIC), Facultat de Farmàcia and Institut de Biomedicina (IBUB), Universitat de Barcelona, Av. Joan XXIII, 27-31, Barcelona E-08028, Spain; E-Mails: irenesola872@gmail.com (I.S.); elisabet.viayna@gmail.com (E.V.); tania.gomez.nadal@gmail.com (T.G.); cargalcan@hotmail.com (C.G.); matteo.cassina01@universitadipavia.it (M.C.); camps@ub.edu (P.C.); 2 Department of Molecular Biochemistry and Pharmacology, IRCCS-Istituto di Ricerche Farmacologiche “Mario Negri”, Via La Masa 19, Milan 20156, Italy; E-Mails: margherita.romeo@marionegri.it (M.R.); luisa.diomede@marionegri.it (L.D.); mario.salmona@marionegri.it (M.S.); 3 Chiral Technologies Europe, Parc d’Innovation, Bd. Gonthier d’Andernach, Illkirch F-67400, France; E-Mails: pfranco@chiral.fr (P.F.); mschaeffer@chiral.fr (M.S.); dcolantuono@chiral.fr (D.C.); drobin@chiral.fr (D.R.); 4 Neuropharmacology Department of QPS Austria-Gmbh, Parkring 12, Grambach 8074, Austria; E-Mails: Daniela.Brunner@qps.com (D.B.); Nicole.Taub@qps.com (N.T.); Birgit.Hutter-Paier@qps.com (B.H.-P.)

**Keywords:** disease-modifying anti-Alzheimer drugs, multitarget drugs, neuroprotection, animal models of Alzheimer’s disease, multigram preparative chromatographic resolution

## Abstract

We describe the multigram synthesis and *in vivo* efficacy studies of a donepezil‒huprine hybrid that has been found to display a promising *in vitro* multitarget profile of interest for the treatment of Alzheimer’s disease (AD). Its synthesis features as the key step a novel multigram preparative chromatographic resolution of intermediate racemic huprine Y by chiral HPLC. Administration of this compound to transgenic CL4176 and CL2006 *Caenorhabditis elegans* strains expressing human Aβ42, here used as simplified animal models of AD, led to a significant protection from the toxicity induced by Aβ42. However, this protective effect was not accompanied, in CL2006 worms, by a reduction of amyloid deposits. Oral administration for 3 months to transgenic APP_SL_ mice, a well-established animal model of AD, improved short-term memory, but did not alter brain levels of Aβ peptides nor cortical and hippocampal amyloid plaque load. Despite the clear protective and cognitive effects of AVCRI104P4, the lack of Aβ lowering effect *in vivo* might be related to its lower *in vitro* potency toward Aβ aggregation and formation as compared with its higher anticholinesterase activities. Further lead optimization in this series should thus focus on improving the anti-amyloid/anticholinesterase activity ratio.

## 1. Introduction

Alzheimer’s disease (AD) currently constitutes a huge human tragedy due to its devastating effects on the quality of life of patients, and its increasingly higher prevalence and mortality. AD currently affects some 44 million people worldwide and is among the top ten leading causes of death in developed countries, and the trend is that these figures will keep on increasing in the next decades [1].

The drugs marketed so far to combat AD were developed to alleviate the symptoms of the disease, *i.e.*, the cognitive and functional decline mainly caused by a cholinergic deficit in the central nervous system (CNS). As long as more players in the neuropathogenesis of AD, placed upstream of the neurotransmitter deficits that originate AD symptomatology, have been known, intensive research has been conducted to develop new drugs able to modulate those upstream targets and, hence, the underlying mechanisms of the disease. A number of promising drug candidates have been developed in the past decade to separately hit one of the putative key players of AD neuropathogenesis, prominently the formation or the aggregation of the β-amyloid peptide (Aβ) [2,3]. However, none of these single-target disease-modifying agents has demonstrated to be effective and safe in clinical trials, so far. In this context, alternative approaches are worth of consideration in the pursuit of effective drugs that may prevent, stop or delay AD progression. An alternative approach, which is gaining adepts in the last years, is based on the consideration of AD as a pathological network where several protein targets of similar relevance are interconnected, leading to a robust system that cannot be perturbed by separate modulation of any single target [4]. The therapeutic strategy that follows from this novel conception of the disease is evident, *i.e.*, the simultaneous modulation of several important targets of the disease network seems to be a more realistic option for tackling the neurodegenerative process of AD [5,6,7,8,9].

Indeed, this approach has proved successful in recent *in vivo* studies with some multitarget compounds such as memoquin [10] and IQM-622 [11] in different mouse models of AD, where these compounds have been shown to be able to address the underlying mechanisms of neurodegeneration.

**Scheme 1:**
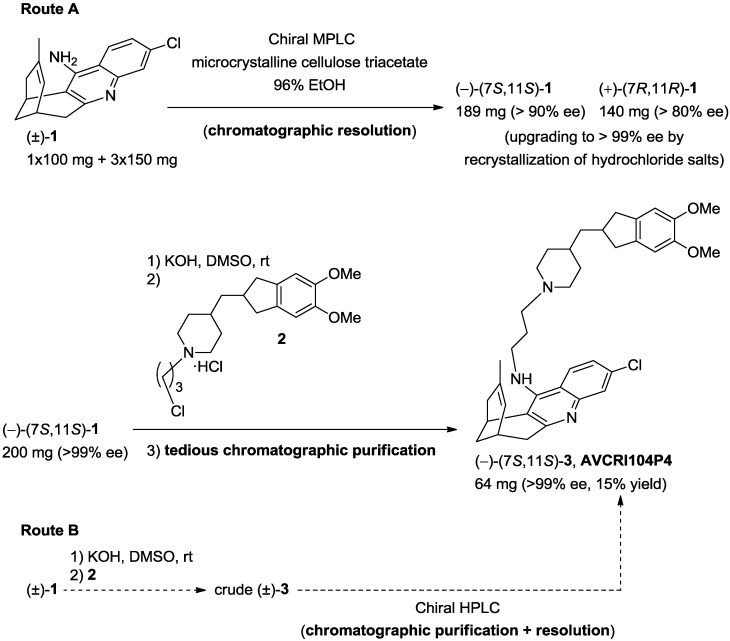
Reported low scale synthesis of AVCRI104P4 (route A) and envisaged alternative sequence (route B).

We have recently developed a new structural family of hybrid compounds, which were designed by combination of pharmacophoric moieties of two potent inhibitors of acetylcholinesterase (AChE), namely donepezil and huprine Y [12]. The rationale behind this design was to enable interactions at different sites of a particular target, simultaneously affecting two important targets in the context of AD treatment. Thus, the novel donepezil‒huprine hybrids were decorated with structural motifs as to enable their simultaneous interactions at three different sites all along the 20 Å-deep catalytic gorge of the enzyme AChE [13], namely the active, midgorge and peripheral sites, which should lead to a potent cholinergic effect. On the other hand, this multiple-site binding to AChE was expected to lead to a disruption of the binding of the AChE peripheral site to Aβ [14,15], and hence, to an inhibition of the Aβ proaggregating action of AChE [16,17]. Indeed, *in vitro* these donepezil-huprine hybrids were endowed with both activities for which they had been rationally designed. Additionally, screening at other targets of interest for AD treatment revealed that these hybrids can also inhibit *in vitro* butyrylcholinesterase (BChE), Aβ self-aggregation and BACE-1, the enzyme that catalyzes the first and rate-limiting step of the proteolytic cleavage of the amyloid precursor protein (APP) to Aβ, thereby expanding the *in vitro* multitarget profile of these compounds. AVCRI104P4 (Scheme 1) emerged as the lead of this structural family, by virtue of its interesting *in vitro* multitarget profile (Table 1).

**Table 1:** *In vitro* and *ex vivo* biological profile of AVCRI104P4 relative to the parent compounds donepezil and huprine Y [12].

Compound	hAChE IC_50_ nM	hBChE IC_50_ nM	BACE-1 IC_50_ µM	Aβ Aggreg. % Inhibition at 10 µM ^a^	PAMPA-BBB *P*_e_ (10^‒6^ cm·s^‒1^) ^b^ (Prediction)	*Ex Vivo* Studies ^c^ % Inhibition Brain AChE
AVCRI104P4	2.61	349	11.0	29	11.4 (CNS+)	59% at 5 min ^d^
Donepezil	21.4	7273	11.3	<5	25.2 (CNS+)	73% at 5 min ^e^
(‒)-Huprine Y	0.43	247	Nd ^f^	10.2	18.2 (CNS+)	97% at 20 min

^a^ [Aβ1‒42]/[inhibitor] = 5:1; ^b^ Permeability (*P*_e_) results from the PAMPA-BBB assay [18], AVCRI104P4 dissolved in PBS/EtOH 70:30, donepezil and (‒)-huprine Y dissolved in PBS/EtOH 80:20; ^c^ % Inhibition of OF1 mice brain AChE at the indicated time after i.p. administration of 10 µmol·kg^‒1^ of the compound *versus* untreated controls; ^d^ 46% at 20 min (unpublished results); ^e^ 68% at 20 min; ^f^ Not determined, 14% inhibition at 5 µM.

It is generally believed that multitarget compounds should display similar *in vitro* potencies at their different biological targets, usually within one order of magnitude of each other, which might lead to a similar level of occupancy of those targets *in vivo* [19]. However, it has been suggested that the potencies at the different targets may not necessarily have to lie within such a narrow range, so that *in vivo* studies of a lead candidate may be very helpful to ascertain the adequate ratio of *in vitro* activities [19]. Thus, even though the two main *in vitro* activities of AVCRI104P4 might seem not properly balanced, with anticholinesterase activities in the nanomolar (AChE) to submicromolar (BChE) range and anti-amyloid activity (BACE-1) in the low micromolar range, *in vivo* testing of this compound was envisaged to definitely find out whether the different activities of this lead might arise also *in vivo*. 

Herein, we describe the synthesis at a multigram scale of AVCRI104P4, which involves as the key step a novel multigram preparative chromatographic resolution of the intermediate racemic huprine Y by chiral HPLC, and the *in vivo* efficacy studies of AVCRI104P4 in different animal models of AD, two transgenic *Caenorhabditis elegans* strains expressing Aβ42 (CL4176 and CL2006) as simplified invertebrate models, and transgenic APP_SL_ mice, as a well-established animal model. Particularly, the protective effects of AVCRI104P4 against the paralysis induced in CL4176 by the oligomeric production of Aβ1‒42 and in CL2006 by the constitutive expression of Aβ3‒42, leading to both oligomeric and fibrillar protein deposition, have been assessed. Moreover, the behavioural effects of AVCRI104P4 in APP_SL_ transgenic mice orally treated for 3 months, as well as its effects on Aβ levels in cerebrospinal fluid (CSF) and brain homogenates, and on the amyloid load in cortex and hippocampus have been also determined.

## 2. Results and Discussion

### 2.1. Synthesis of AVCRI104P4

AVCRI104P4 had been previously synthesized at a centigram scale (64 mg) [12]. However, the planned *in vivo* studies required multigram amounts of this compound, thereby making it necessary a scale-up the synthesis.

The low scale synthesis of AVCRI104P4 involved the preparative chromatographic resolution of racemic huprine Y (**1**), the synthesis of the donepezil-derived chloropropylpiperidine **2**, and the final coupling of enantiopure (–)-(7*S*,11*S*)-huprine Y with **2** (route A in Scheme 1) [12]. However, because purification of AVCRI104P4 by standard silica gel column chromatography had been a quite difficult, low yield, and tedious task in the low scale synthesis [12], a quite attractive option for the scale-up synthesis might involve the direct preparative chiral chromatography of multigram amounts of the crude racemic final compound, inasmuch as separation of AVCRI104P4 from its enantiomer and from unreacted starting materials or other byproducts formed in the reaction might be done at once, thereby avoiding a previous achiral chromatographic step (route B in Scheme 1). Thus, a crucial decision before starting the scaled up synthesis of AVCRI104P4 was the step at which the chromatographic resolution should be done.

In this context, samples of pure racemic **1**, pure racemic **3**, and crude racemic **3** were subjected to an exhaustive screening in liquid and supercritical fluid chromatography (LC and SFC), in order to identify suitable preparative methods and subsequently compare them.

A number of chiral stationary phases (CSPs) were able to separate both racemates for analytical purposes. Nevertheless, for the preparative application, the first important preliminary observation was the limited solubility of the three samples in many organic solvents. Therefore, the screening of mobile phases had to be dictated by the solubility, otherwise, the preparative resolution would be inefficient. Dichloromethane (DCM), EtOAc and THF mixtures were chosen as preferred options and this was conditioning the type of CSPs to be used.

The three samples were screened on polysaccharide-derived columns, having the chiral selector immobilized onto the silica backbone. Those phases (CHIRALPAK IA, IB and IC) were combining a high loading capacity with the possibility of using the above mentioned solvents as mobile phases and/or injection solvents [20,21,22]. Separations for the enantiomeric pairs were identified in LC and SFC mode, however, LC seemed to be better adapted to these molecules.

One of the main advantages of SFC at preparative scale is the possibility of working in CO_2_ mixtures with a relatively low percentage of co‑solvent (usually 10%–20%). However, both **1** and **3** were rather retained in SFC mode, so that higher co‑solvent percentages were needed and solubility was an issue. Therefore, the SFC option was quickly discarded.

Taking into account all these elements, the screening undertaken in LC was reviewed and put into perspective. The best option found was the resolution of the racemic precursor, huprine Y, (±)-**1**, on CHIRALPAK IC with a mobile phase composed by a DCM/i-PrOH mixture (details may be seen in the Experimental Section). The main reasons for such a decision were: (i) the impossibility of isolating (–)‑(7*S*,11*S*)-**3** (AVCRI104P4) in a single chromatographic step from the crude material and (ii) the low solubility of the molecules.

**Figure 1:**
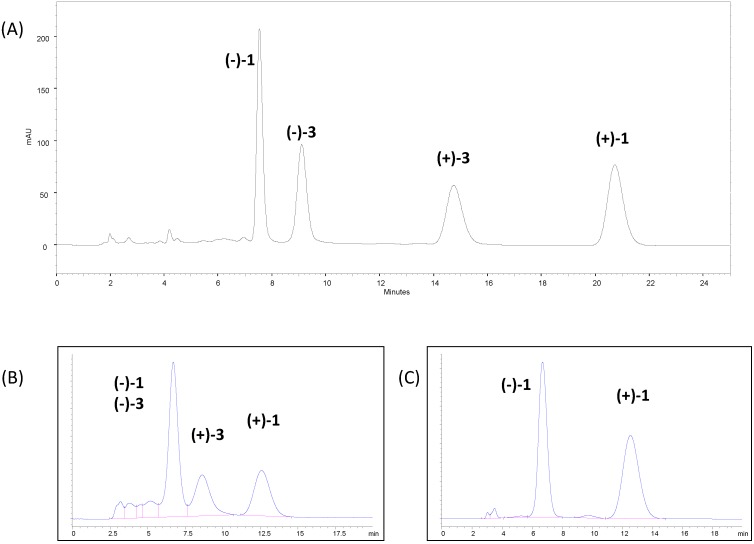
Chromatographic separation by HPLC of (**A**) crude mixture of racemic **1** and **3** on CHIRALPAK IC (5 µm, 250 × 4.6 mm) in *n*‑heptane/DCM/EtOH/DEA 50:50:1:0.1, 2 mL/min, 25 °C; (**B**) crude mixture of racemic **1** and **3** on CHIRALPAK IC (20 µm, 250 × 4.6 mm) in DCM/i‑PrOH/DEA 95:5:0.1, 1 mL/min, 25 °C; (**C**) Racemic **1** on CHIRALPAK IC (20 µm, 250 × 4.6 mm) in DCM/i‑PrOH/DEA 95:5:0.1, 1 mL/min, 25 °C.

In Figure 1A the initially identified resolution of the peaks in the crude material at analytical level (with peak identification) can be found. Such conditions involved 50% of *n*‑heptane in the mobile phase and the sample was only sparingly soluble. It was necessary then optimizing the method by avoiding the alkane and modulating retention, as well as recognition with a DCM/i-PrOH mixture (see chromatogram of the crude in Figure 1B). The combination of DCM with other solvents such as THF was tested, but found to be less adapted. In DCM/i-PrOH, (–)‑**3** (AVCRI104P4) and (–)‑**1** ((–)‑huprine Y)) co‑elute and the two enantiomers of **3** hardly reach baseline separation. Therefore, it seemed that the preparative resolution of racemic huprine Y, (±)-**1**, was the best option (Figure 1C).

Intermediate racemic huprine Y (±)-**1**, and chloroderivative **2** were synthesized at multigram scale following the described procedures [12,23,24,25,26,27], with some necessary modifications due to the scale of work (see Supplementary Materials).

At this point, taking into account the results of the chromatographic screening (Figure 1), we undertook the multigram chromatographic resolution of intermediate huprine Y, (±)-**1**. As outlined in Scheme 1, we had performed the low scale synthesis of enantiopure huprines by preparative chromatographic resolution of the corresponding racemates, using a medium pressure liquid chromatography (MPLC) equipment, microcrystalline cellulose triacetate as the chiral stationary phase and 96% EtOH as the eluent [26]. Indeed, we had separated (–)-(7*S*,11*S*)-huprine Y (189 mg, >90% ee) and (+)-(7*R*,11*R*)-huprine Y (140 mg, >80% ee) using this methodology, after four successive injections of the racemic compound (1 × 100 mg + 3 × 150 mg) [26]. The enantiomeric excesses were subsequently upgraded upon conversion into the corresponding hydrochloride salts followed by recrystallization. Obviously, this laboratory equipment and scale of work were by far insufficient to perform the chromatographic resolution of decagram amounts of racemic huprine Y.

**Scheme 2:**
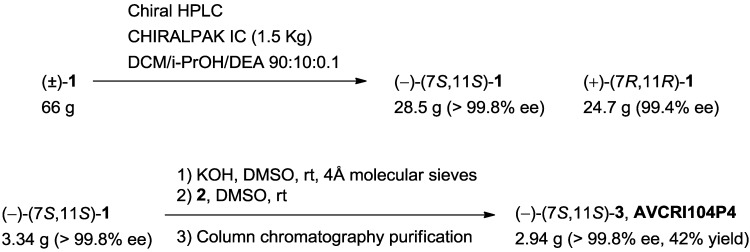
Novel multigram HPLC resolution of huprine Y and scale-up synthesis of AVCRI104P4.

The large scale chromatographic resolution of huprine Y was carried out by preparative HPLC on a 66 g batch (scale factor ×120 relative to the low scale synthesis) using a CHIRALPAK IC column (cellulose tris(3,5-dichlorophenylcarbamate)) and a mixture DCM/i‑PrOH/DEA 90:10:0.1 as the eluent, to yield >99% ee (–)-(7*S*,11*S*)-huprine Y (28.5 g) and (+)-(7*R*,11*R*)-huprine Y (24.7 g) (Scheme 2). The estimated throughput would be 72 g of (–)-(7*S*,11*S*)-huprine Y per day using a 250 × 110 mm‑column in a 24/24 h process by batch chromatography (144 g of racemate processed), *i.e.*, 96 g of racemate per kg of CSP and day. If such a method were translated to continuous chromatography (Simulated Moving Bed, SMB) it would involve about 300 g of pure enantiomer per kg of CSP and day.

Overall, the multigram chromatographic resolution of huprine Y was performed not only with the necessary much higher loading capacity but also with higher enantiopurity and resolution efficiency, so that amounts of both enantiomers >150-fold larger than those obtained in the low scale synthesis were obtained.

Finally, reaction of enantiopure (–)-(7*S*,11*S*)-huprine Y (3.34 g, >99.8% ee) with chloroderivative **2** in the presence of KOH in DMSO afforded AVCRI104P4 (2.94 g) in significantly higher yield than in the low scale procedure (42% isolated yield, after silica gel column chromatography purification *vs.* 15% isolated yield in the low scale synthesis, Scheme 2). After several runs of this reaction, a total amount of 14 g of AVCRI104P4 was finally obtained, which allowed us to undertake the planned *in vivo* studies.

### 2.2. In Vivo Studies in C. elegans

Before testing AVCRI104P4 in a mouse model of AD, we conducted some *in vivo* studies in a simplified invertebrate model, namely in *Caenorhabditis elegans*. This nematode offers a number of advantages such as a short lifecycle, high reproduction profile, experimental flexibility, and ease to knockdown gene expression, among others, which has prompted the creation of different strains that express amyloidogenic proteins involved in different neurodegenerative disorders [28]. Thus, transgenic *C. elegans* AD models have been used to study pathogenic pathways and alterations of gene expression induced by Aβ [29,30], and, recently, also for the evaluation of the neuroprotective and anti-amyloidogenic effects of some multitarget anti-Alzheimer compounds [31,32,33].

Herein, we determined the ability of AVCRI104P4 to protect from the toxicity induced by human Aβ42 expression and accumulation, which results in plaque deposits similar to those observed in the brain of AD patients [34]. To this end, the transgenic *C. elegans* strain CL2006, constitutively expressing human Aβ3–42 in the body wall muscle cells [35,36], was here used. This strain is characterized by a progressive and age-related reduction of the motility due to the expression of Aβ3–42 which results in the formation of both fibrils and oligomers [36]. The CL4176 transgenic *C. elegans* strain, in which the human Aβ1–42 expression was induced by increasing the temperature of culture, was also used. In this strain, the paralysis phenotype is specifically associated with the deposition of oligomers in their muscle cells without the formation of amyloid aggregates [36,37]. Previous results obtained in CL4176 nematodes treated with anti-amyloidogenic tetracyclines supported the toxic role of oligomers [36,37,38]. CL802 nematodes, which do not express the Aβ transgene [34], were used as controls.

**Figure 2:**
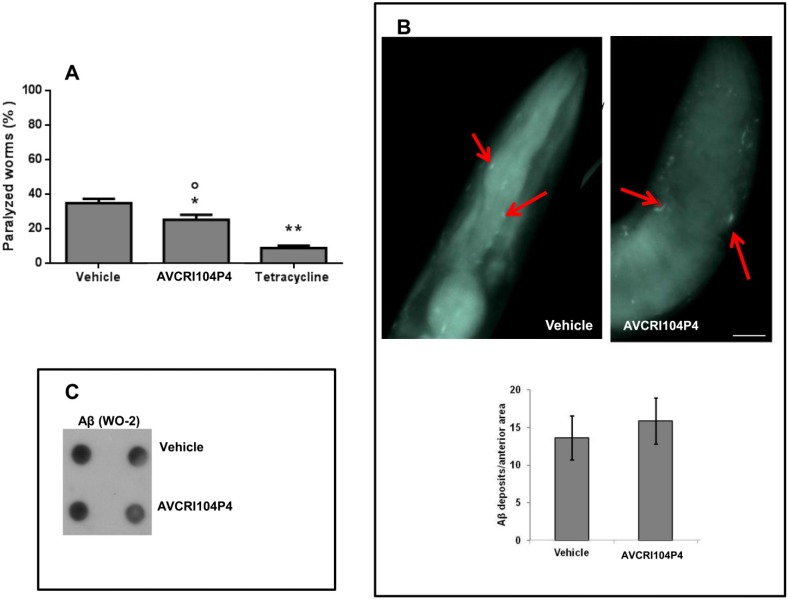
Effect of AVCRI104P4 administration on the paralysis of CL2006 nematodes and Aβ deposition. (**A**) Percentage of CL2006 paralyzed worms treated with AVCRI104P4. Nematodes were treated for 24 h with 100 µM AVCRI104P4 or 100 µM tetracycline (positive control) and the number of nematodes that did not move was scored. Data from three independent experiments (*n* = 100, per group) are indicated as % ± S.D. of paralyzed nematodes to worms treated with vehicle. * *p* < 0.05 and ** <0.01 *vs.* vehicle-treated CL2006 nematodes and ° *p* < 0.05 *vs.* CL2006 worms treated with tetracycline (One-way ANOVA test); (**B**) X-34 positive amyloid deposits in CL2006 worms treated with vehicle or 100 µM AVCRI104P4. Synchronized CL2006 nematodes were treated with vehicle or 100 µM AVCRI104P4 for 24 h, amyloid plaques were stained with X-34 dye and visualized at short wavelength excitation. Scale bar 20 µm. Amyloid deposits in the anterior area of worms, indicated with red arrows, were quantified in nematodes treated with vehicle (*n* = 20) or 100 µM AVCRI104P4 (*n* = 20), by counting the number of X-34 positive spots; (**C**) Effect of 100 µM AVCRI104P4 administration on total Aβ in CL2006 worms. Representative dot blot of total Aβ (WO2) on lysates (5 µg) of CL2006 transgenic worms treated with vehicle or 100 µM AVCRI104P4 for 24 h.

In CL2006 worms the administration of 100 µM AVCRI104P4 for 24 h caused a little, albeit significant, reduction of 26.5% of the Aβ3–42-induced paralysis, significantly lower than that occurring with 100 µM tetracycline (74.6% reduction of paralysis) (Figure 2A). The feeding of CL802 worms with 100 µM AVCRI104P4 for 24 h did not modify their paralysis (2.3% ± 1.0% and 2.7% ± 0.9% of percentage of paralyzed worms for CL802 nematodes fed with vehicle and AVCRI104P4, respectively), with this result being indicative of the fact that this compound did not induce any unspecific and/or toxic effect in control nematodes.

We then investigated the effect of AVCRI104P4 on the degree of Aβ amyloidosis by evaluating the number of aggregate deposits in the head region of CL2006 transgenic worms. To this end, synchronized worms were grown at 16 °C for 72 h and treated with either vehicle or AVCRI104P4 (100 µM, 100 µL/plate) for 24 h. Nematodes were then stained with the X-34 dye, specifically recognizing β-amyloid deposits [36]. AVCRI104P4 did not reduce the X-34 positive spots (Figure 2B), which indicated that the protective effect of the compound cannot be ascribed to its ability to affect Aβ fibril deposition. The dot blot analysis performed on worm lysates with the WO2 total Aβ-specific antibody (Figure 2C) indicated that there were no differences in the Aβ-immunoreactive signal between worms treated for 24 h, with vehicle or 100 µM AVCRI104P4. These data indicate that the ability of AVCRI104P4 to counteract Aβ toxicity was not linked to its ability to modify the amount of Aβ produced and/or degraded by transgenic nematodes.

**Figure 3:**
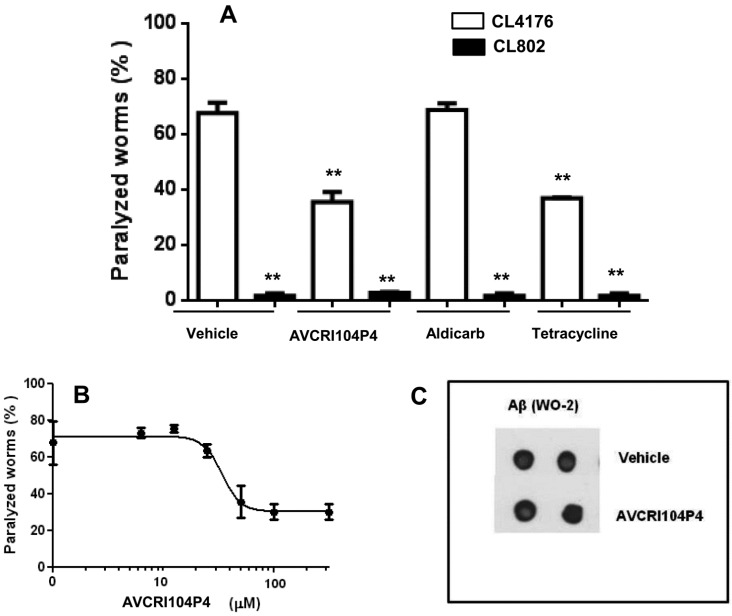
Effect of AVCRI104P4 on the paralysis of CL4176 worms. (**A**) Percentage of paralyzed CL4176 and CL802 worms fed with 100 µM AVCRI104P4, 100 µM aldicarb, or 100 µM tetracycline 18 h after the temperature increase. Paralysis was evaluated 42 h after the temperature rise. Data from three independent experiments (*n* = 100, per group) are indicated as % ± S.D. of paralyzed nematodes to worms treated with vehicle. ** *p* < 0.01 *vs.* vehicle-treated CL4176 nematodes (One-way ANOVA test); (**B**) Dose-dependent protective effect of AVCRI104P4 on the paralysis of CL4176 worms. The transgene expression was induced in synchronized worms after 54 h at 16 °C, by raising the temperature to 24 °C. Nematodes were fed with different concentrations of AVCRI104P4 (5–300 µM, 100 µL/plate) 18 h after raising the temperature. The number of paralyzed worms was scored 42 h after the temperature rise. Data from three independent experiments (*n* = 100, per group) are indicated as % ± S.D. of paralyzed nematodes to worms treated with vehicle; (**C**) Effect of 100 µM AVCRI104P4 administration on total Aβ in CL4176 worms. Representative dot blot of total Aβ (WO2) on lysates (5 µg) of CL4176 transgenic worms treated with vehicle or 100 µM AVCRI104P4 as described.

The effect of AVCRI104P4 on CL4176 worms was also investigated since this strain represents a convenient model to specifically study the *in vivo* effects of Aβ oligomers and to screen for potential anti-oligomer compounds [35,36]. Administration of 100 µM AVCRI104P4, 18 h after the rise of temperature, reduced the paralysis by 47.5%, similarly to 100 µM tetracycline (45.4% reduction of paralysis) (Figure 3A). Aldicarb, an AChE inhibitor, did not exert any protective action (Figure 3A), indicating that the protective activity of AVCRI104P4 against the toxicity exerted by oligomeric Aβ was specific and unrelated to the inhibition of AChE. In addition, AVCRI104P4 protected CL4176 nematodes from the Aβ-induced paralysis in a dose-dependent manner (Figure 3B). The drug concentration that inhibited paralysis by 50% (IC_50_) was 34.0 ± 1.2 µM (*n* = 100 worms/group, mean ± SE), similar to that obtained for tetracycline (39.9 ± 1.5 µM) [36].

The protective effect of AVCRI104P4 in CL4176 worms was not associated to its ability to modify the amount of Aβ produced by transgenic nematodes, as indicated by the dot blot analysis performed with the WO2 total Aβ-specific antibody (Figure 3C). These results indicate that the protective effect of AVCRI104P4 against Aβ toxicity was greater in CL4176 than in CL2006 nematodes, suggesting that this drug may exert its activity against the oligomeric assemblies of Aβ, without affecting amyloid plaque deposition and independently from its AChE inhibitory activity.

### 2.3. In Vivo Studies in Transgenic APP_SL_ Mice

The effects of AVCRI104P4 on cognition and amyloid pathology were evaluated *in vivo* in a well-established animal model of AD, namely in APP_SL_ transgenic mice. These transgenic mice develop early and progressive amyloid plaque deposition in cortex and hippocampus as well as cognitive impairment, thereby being widely used as preclinical animal models of AD. Twenty-four hAPP_SL_ transgenic animals were treated orally with vehicle only or AVCRI104P4 daily for 3 months. Starting with a dose of 10 mg/kg of AVCRI104P4, the treatment dose was increased in a staggered manner, *i.e.*, weekly + 5 mg/kg, up to a final dosage of 40 mg/kg per day.

#### 2.3.1. Behavioral Studies

**Figure 4:**
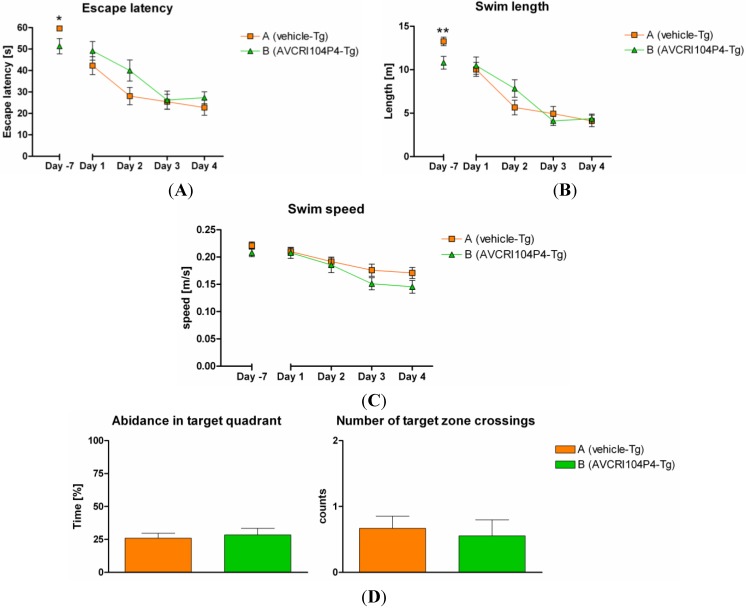
Graph represents (**A**) the latency to escape from the water (escape latency); (**B**) the swimming path to escape from the water (swim length); (**C**) the swim speed; and (**D**) the abidance in the target quadrant and number of target zone crossings in the MWM of hAPP_SL_ transgenic animals treated with vehicle only (group A, orange) or AVCRI104P4 (group B, green) for 3 months. Data are presented as mean ± SEM for each treatment group and day. Statistical significant differences were detected on the very first training day (-7) using Mann-Whitney test (for (A)) or unpaired t-test (for (B)). * indicates *p* < 0.05. ** indicates *p* < 0.01. No statistical significant differences were detected for (C) and (D).

The Morris Water Maze (MWM) was performed at the end of the treatment period to evaluate the effects of AVCRI104P4 on cognition. During the last week of treatment, we planned the evaluation of the spatial navigation learning and memory of animals by assessing the escape latency (the time (s) the mice needed to find the hidden platform and therefore to escape from the water), the swim length (the length of the trajectory (m) to reach the target), the swim speed (calculated quotient of swim length and escape latency) and the average distance to the target zone (m), as well as the number of target crossings and the abidance in the target quadrant during the probe trial. On the first MWM training day (day -7), some compound treated animals had problems to keep their head above the water, which might be ascribed to an overdosing of AVCRI104P4. Despite this handicap, AVCRI104P4 treated animals performed significantly better in terms of escape latency and swim length compared to vehicle treated controls. Indeed, animals treated with AVCRI104P4 found the hidden platform in a significantly shorter time (Figure 4A) and needed a significantly shorter way to find the hidden platform (Figure 4B) than animals receiving vehicle only. These results indicated a clear improvement of the short-term memory upon treatment with AVCRI104P4.

After a washout period of 5 days, the MWM test was repeated. Learning curves of days 1 to 4 of the postponed MWM training phase are depicted in Figure 4. During the main training phase, animals of both groups showed typical declining learning curves in terms of escape latencies and swimming paths. However, mice that had been treated with AVCRI104P4, which had shown improved short-term memory on the first MWM training day (day -7), did not show significantly improved spatial learning capabilities relative to controls after the washout period, *i.e.*, in the absence of treatment, neither on day 1 nor on the following days (Figure 4A,B, and Figures S1 and S2, Supplementary Materials).

Similarly, no significant differences between treatment groups were detected when calculating the swimming speed (Figure 4C), the time spent in the target quadrant where the platform was hidden (abidance) and the number of target zone crossings (Figure 4D).

Thus, AVCRI104P4 treated animals started with significant lower escape latency and shorter way to find the hidden platform on training day -7, when training was assessed short after the last application of 40 mg/kg AVCRI104P4, but its clear cognitive effects disappeared from training day 1 onwards, after the washout period, likely because these mice did not receive any compound for 5 days.

#### 2.3.2. Biochemistry and Histology

Aβ38, Aβ40, and Aβ42 levels were measured with an immunosorbent assay in CSF samples as well as in two brain homogenate preparations of transgenic mice, in which brain proteins from the left hemisphere of transgenic animals were extracted with diethylamine (DEA), which solubilizes non-plaque associated Aβ such as monomeric to oligomeric structures or with formic acid (FA), which dissolves the total Aβ in the brain homogenates including plaques. Also, amyloid load was visualized with 6E10 antibody and thioflavin S (ThioS) staining and the signal was quantitatively analyzed in terms of the number of plaques, their total surface area, and their average size, both in the cortex and hippocampus.

Unfortunately, treatment with AVCRI104P4 did not alter Aβ38, Aβ40, and Aβ42 levels, neither in the CSF nor in DEA and FA brain homogenate preparations (Figure S3, Supplementary Materials), nor the number, area and mean size of 6E10 or ThioS objects in cortex and hippocampus (Figures S4–S6, Supplementary Materials) compared to vehicle treated controls. Similarly, plaque morphology, conformation of diffusely aggregated 6E10 immunoreactive amyloid and dense ThioS positive cores, and overall intracellular somal amyloid labeling was in general comparable between vehicle and AVCRI104P4 treatment groups (Figures S7‒S9, Supplementary Materials).

Thus, contrary to our expectations, the moderately potent BACE-1 inhibitory activity observed *in vitro* for AVCRI104P4 did not result in any anti-amyloidogenic effect *in vivo* in hAPP_SL_ transgenic mice. Conversely, its potent *in vitro* anti-cholinesterase activity leads to a clear cognitive enhancing effect in transgenic mice, which disappears in the absence of treatment. All together, the results obtained in the *in vivo* efficacy studies point to a purely cholinergic but non disease-modifying effect of AVCRI104P4, thereby highlighting the need to improve its *in vitro* anti-amyloid/anticholinesterase activity ratio in future lead optimization.

### 2.4. Hepatotoxicity Studies in HepG2 Cells

**Figure 5:**
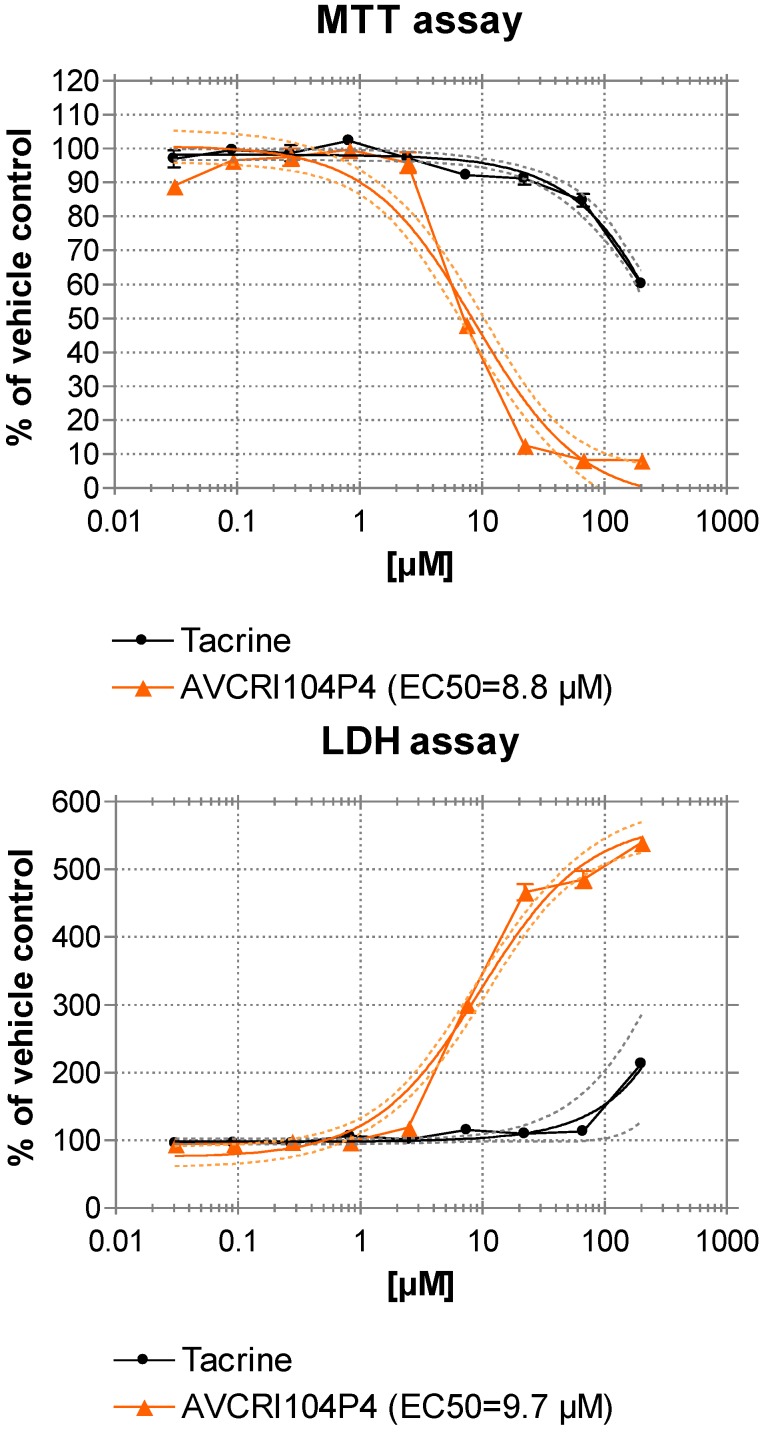
AVCRI104P4 and tacrine dose response in HepG2 cells. HepG2 cells were treated with AVCRI104P4 and tacrine at the indicated concentrations for 24 h. Cell viability and cellular toxicity were determined according to MTT and LDH assays, respectively. Values are shown as mean ± SEM (*n* = 6).

The huprine Y moiety of AVCRI104P4 is structurally related to tacrine, the first approved anti-Alzheimer drug which was withdrawn from the market due to its hepatotoxic effects. Despite the large number of tacrine-based multitarget compounds or structurally related derivatives that are being developed, very few reports about their potential hepatotoxicity can be found in the literature. To address this issue in this work, the effects of AVCRI104P4 on cell viability and cytotoxicity were determined by MTT and LDH assays in HepG2 cells, a human liver hepatocellular carcinoma cell line, and compared with those produced by tacrine. HepG2 cells were incubated with rising dose concentrations of AVCRI104P4 and tacrine (0, 0.03, 0.1, 0.3, 0.8, 2.5, 7.4, 22.2, 66.7, and 200 µM). HepG2 cells showed a cytotoxic response to increasing dose concentrations of AVCRI104P4, which exhibited EC_50_ values of 8.8 and 9.7 µM in the MTT and LDH assays, respectively (Figure 5). In the case of tacrine, minor toxic effects started at concentrations higher than 66.7 µM but its EC_50_ values could not be determined since toxic concentrations were beyond the indicated concentration range. Hence, notwithstanding the apparent lack of toxicity of other huprine derivatives in chronic *in vivo* studies in different mouse models, we have found that AVCRI104P4 is more toxic to HepG2 cells than tacrine itself. Of note, similar results have been found for bis(7)-tacrine, an heptamethylene-linked dimer of tacrine under preclinical development that features a similar multitarget profile to that of AVCRI104P4 [39], for which hepatotoxicity in mice [40] and a higher cytotoxicity than that of tacrine in Caco-2 cells [41] have been recently reported.

## 3. Experimental Section

### 3.1. Synthesis of AVCRI104P4

#### 3.1.1. Chromatographic Screening

The screening was performed on CHIRALPAK IA, CHIRALPAK IB and CHIRALPAK IC (250 × 4.6 mm i.d., 5 μm) supplied by Daicel Corporation (Tokyo, Japan) in LC and SFC. Flow rates in LC were set at 1 or 2 mL/min (depending on retention) and temperature at 25 °C. The SFC screening was performed with MeOH, EtOH, and i-PrOH as co-solvents in CO_2_ and extended to other polysaccharide-derived columns. The LC screening covered *n*-heptane mixtures with alcohols, THF, EtOAc, DCM, as well as pure acetonitrile and alcohols, with a number of combinations to optimise selectivity, retention and solubility. Optimisations were performed on the corresponding 20 μm materials.

#### 3.1.2. Chromatographic Resolution of (±)-Huprine Y at Preparative Scale

Racemic huprine Y (66 g) was resolved into its two enantiomers using DCM/i-PrOH/DEA 90:10:0.1 as the mobile phase. The chiral stationary phase was ca. 1.5 Kg of CHIRALPAK IC (20 μm), available from Daicel Corporation, packed into a 250 × 110 mm column. (–)-(7*S*,11*S*)-Huprine Y, (–)-(7*S*,11*S*)-**1**, was the first eluted enantiomer with this chromatographic method and its enantiomeric excess was >99.8%. Evaporation to dryness of the solvent yielded a thick oily material, which could be transformed into a solid by subsequent dissolution in DCM and evaporation. A total amount of 28.5 g of (–)-(7*S*,11*S*)-**1** was isolated as a brown amorphous powder and used for the next reaction without further purification. The non-target (second eluting peak, (+)-(7*R*,11*R*)-**1**, 24.7 g) was also recovered at high % ee (99.4%) to keep the overall high recovery.

#### 3.1.3. (–)-(7*S*,11*S*)-3-Chloro-12-[(3-{4-[(5,6-dimethoxyindan-2-yl)methyl]piperidin-1-yl}propyl) amino]-6,7,10,11-tetrahydro-9-methyl-7,11-metanocycloocta[*b*]quinoline, AVCRI104P4 [(–)-(7*S*,11*S*)-**3**]

A suspension of (–)-huprine Y, (–)-(7*S*,11*S*)-**1** (>99.8% ee, 3.34 g 11.7 mmol) and finely powdered KOH (85% purity, 3.90 g, 58.5 mmol) in anhyd. DMSO (48 mL) was stirred, heating every 10 min approximately with a heat gun for 1 h and at room temperature for an additional 1 h, and treated dropwise with a solution of crude **2** (5.00 g of a crude that could contain a maximum of 14.1 mmol of **2**, see Supplementary Materials) in anhydrous DMSO (40 mL) (previously warmed at 70 °C). The reaction mixture was stirred at room temperature for 3 days, diluted with H_2_O (150 mL), and 10 N NaOH (100 mL), and extracted with DCM (3 × 200 mL). The combined organic extracts were washed with H_2_O (2 × 200 mL), dried over anhydrous Na_2_SO_4_, and evaporated under reduced pressure to give a brown solid (10.9 g), which was purified through column chromatography (35–70 μm silica gel, EtOAc/hexane/Et_3_N 50:50:0.2), affording successively unreacted (–)-huprine Y (579 mg) and AVCRI104P4 (2.94 g, 42% yield). The IR, ^1^H- and ^13^C-NMR data coincided with those previously reported [12]; Anal. calcd for C_37_H_46_ClN_3_O_2_·2HCl·1.75H_2_O: C 63.06%, H 7.37%, N 5.96%, Cl 15.09%, found: C 63.16%, H 7.33%, N 5.49%, Cl 14.80%. After several runs of this reaction, a total amount of 14 g of AVCRI104P4 was obtained.

### 3.2. In Vivo Studies in Caenorhabditis elegans

#### 3.2.1. *C. elegans* Strains

CL2006, CL4176 and the control CL802 transgenic strains were obtained from the *Caenorhabditis* Genetic Center (University of Minnesota, Minneapolis, MN, USA) [34,42]. CL2006 constitutively expresses Aβ3–42 in the body-wall muscles, while Aβ1–42 was produced in muscle cells of CL4176 in a temperature-inducible manner. Nematodes were grown at 16 °C on solid Nematode Growth Medium (NGM) seeded with *E. coli* (OP50) for food. Age-synchronized animals were prepared as already described [34,42] and day 1 synchronized worms were cultured on fresh NGM plates at 16 °C.

#### 3.2.2. Paralysis Assay

The paralysis assay was performed in the CL4176 and CL802 worms as already described [35,36] Transgene expression was induced after 54 h of growth at 16 °C by raising the temperature from 16 to 24 °C. Eighteen hours after the temperature shift (L3 larval stage) nematodes were fed with different concentrations of AVCRI104P4 (5–300 µM, 100 µL/plate, 100 worms/plate) or 100 µM aldicarb. The paralysis of worms was determined 42 h after the temperature raise by scoring nematodes that did not move. Fifty µM tetracycline (Fluka, Buchs, Switzerland) administered 18 h after temperature rise was used as positive control. Synchronyzed CL2006 worms were fed, at L3 larval stage, with 100 µM AVCRI104P4 (100 µL/plate, 100 worms/plate) and the number of nematodes paralyzed was scored 24 h later. Tetracycline was administered as a positive control.

#### 3.2.3. Aβ Expression

Transgenic CL2006 and CL4176 worms treated with vehicle or 100 µM AVCRI104P4 were processed as previously described [36]. The proteins were precipitated overnight with MeOH (1:4 *v*/*v*) at −20 °C, before being resuspended in loading buffer and then spotted (5–10 µg) onto nitrocellulose membranes (Merck Millipore, Darmstadt, Germany). Total protein visualization on the blotted membranes was performed by using 0.1% Ponceau Red solution (Sigma Aldrich, Saint Louis, MO, USA). Anti-Aβ mouse monoclonal antibody (1:1000, Clone WO2, Merck Millipore) and peroxidase-conjugated anti-mouse IgG (1:5000, Sigma) were used as the primary and secondary antibodies, respectively. Mean volumes of immunoreactive and Red Ponceau dyed spots were analyzed (Progenesis SameSpots software 4.0, Nonlinear Dynamics Limited Keel House, Newcastle upon Tyne, UK; Nonlinear Dynamics, Newcastle upon Tyne, UK) and the mean values of volume of immunoreactive spot/volume of total Ponceau dyed proteins ± SD were calculated.

#### 3.2.4. Staining of β-Amyloid

Treated and non-treated fixed CL2006 worms [36] were stained with X-34 (1.0 mM) in Tris-HCl (10 mM) for 4 h at room temperature and at pH 8.0 [36,43]. The samples were destained and then observed with a IX-71 Olympus inverted fluorescence microscope, acquiring the images with a CCD camera. Quantification of amyloid burden in the anterior area of vehicle- and AVCRI104P4-treated nematodes (*n* = 20 per group) was carried out through the number of X-34 positive spots and the ratio Aβ deposits/anterior area was calculated.

#### 3.2.5. Statistical Analysis

The effects of AVCRI104P4 on nematodes were compared with those of vehicle by standard tests (independent Student’s *t*-test or One-way ANOVA test) and GraphPad Software (Prism version 4.0 for Windows, San Diego, CA, USA) was used to calculate the IC_50_ values, taking *p* values < 0.05 as statistically significant.

### 3.3. In Vivo Studies in Transgenic APP_SL_ Mice

#### 3.3.1. Animals and Treatment

Male APP_SL_ transgenic mice were housed as previously described [44], *i.e.*, in individual ventilated cages, with constant 12 h light/dark cycle, at 24 °C and 40%–70% humidity, and providing standard rodent chow (Altromin, Lage, Germany) and tap water *ad libitum*. Animals were maintained in the fully AAALAC accredited QPS Austria animal facility under standardized conditions. Animal studies conformed to the Austrian guidelines for the care and use of laboratory animals (BGBl. 501/1989 in the appropriate valid version) and were approved by the Styrian Government, Austria (FA10A-78 Jo 85-2011). Twenty four mice at an age of 6 months (±2 weeks) were orally treated via gavage with vehicle or 40 mg/kg/day of AVCRI104P4 daily for 3 months (*n* = 12 each group). Eight animals were kept in reserve.

#### 3.3.2. Behavioural Studies

The Morris Water Maze (MWM) was performed to assess spatial learning at the end of the treatment period. The MWM consisted of a white circular pool of a diameter of 100 cm, filled with tap water at a temperature of 21 ± 2 °C. Each mouse had to perform three trials on each of four consecutive days, followed by a so-called probe trial (PT) 1 h after the last trial on day 4. Additionally, all animals had to perform a visual test after the PT on the last day to exclude visual problems as reason for behavioural results.

Descriptive statistical analysis was performed on all evaluated parameters. Data were averaged and represented as mean ± standard error of mean (SEM). Outliers detected with Grubb’s test were excluded from data analysis. Normality distribution of the values was tested with Kolmogorov Smirnov normality distribution test. Differences in MWM learning curves were evaluated by a Two-way ANOVA followed by Bonferroni’s post-test. In order not to miss tendencies in the ANOVA, unpaired two-tailed *t*-tests or Mann Whitney tests (if values are not normally distributed) were calculated. Vehicle treated animals served as a control for AVCRI104P4 receiving mice.

#### 3.3.3. Tissue Sampling

Mice were deeply sedated by standard inhalation anesthesia (Isoba^®^, Essex, UK), CSF was collected and the samples were immediately frozen on dry ice and stored at −80 °C until used for Aβ determination. Following transcardial perfusion with 0.9% saline brains were also collected. Cerebellum was cut off and frozen and hemispheres were divided. Left hemispheres were frozen until used for biochemical analysis; right hemispheres for histological investigations were fixed by immersion in a freshly produced 4% mixture of paraformaldehyde/PBS (pH 7.4) for 1 h at room temperature, transferred to a 15% sucrose PBS solution until sunk at 4 °C to ensure cryoprotection, frozen in liquid isopentane, and stored at −80 °C.

#### 3.3.4. Biochemistry

##### Brain Protein Extraction

Left hemi-brain samples without cerebellum of transgenic animals were homogenized and separated into 2 fractions, the formic acid (FA) and diethylamine (DEA) fractions. Brain hemispheres were homogenized in Tissue Homogenization Buffer (THB) (20 mM Tris HCl pH 7.4, 250 mM sucrose; 1 mM EDTA; 1 mM EGTA, protease inhibitor cocktail; 1 mL THB per 100 mg brain tissue). For the extraction of deposited proteins (FA preparation), 200 µL of the THB-homogenate were mixed with 440 µL cold FA and sonicated. 400 µL of the mixture was centrifuged for 1 h at 74,200 *g* at 4 °C and 210 µL supernatant were neutralized with 4 mL FA neutralization solution (1 M TRIS; 0.5 M Na_2_HPO_4_; 0.05% NaN_3_). For the extraction of non-plaque-associated proteins (DEA preparation), 1 mL of the THB-homogenate was mixed with 1 mL DEA solution (0.4% DEA; 100 mM NaCl) and centrifuged for 1 h at 74,200 *g* at 4 °C. 1.7 mL supernatant were neutralized with 170 µL DEA Neutralization Solution (0.5 M Tris HCl, pH 6.8).

##### Determination of Aβ Levels

In the two different brain homogenate fractions (FA and DEA) and in CSF of each transgenic mouse, Aβ38, Aβ40 and Aβ42 levels were measured with a commercially available Aβ-kit from Mesoscale Discovery (Rockville, ND, USA). Samples from the brain preparations were analyzed in duplicate. Due to the small amount, CSF samples were single analyzed only. Aβ levels were evaluated in comparison to peptide standards as ng Aβ per mg brain or ng Aβ per mL CSF. 

#### 3.3.5. Histology

##### Sectioning, Immunohistochemistry and Image Analysis

Seven cryo-sections per medio-sagittal level were sagittally cut at 10 µm slice thickness on a Leica CM 3050S cryotome. Brain levels were chosen. The cut of the twelve levels started with a random section roughly corresponding to Figure 102 in the morphology atlas “The Mouse Brain” (Paxinos and Franklin, 2nd edition; approximately 0.24 mm lateral from midline), then sampling was continued uniformly and systematically, always retaining seven slices per level in series and discarding 230 µm in between the levels.

##### Determination of Plaque Load

Plaque load was determined with 6E10 primary antibody (BioLegend formerly Covance, Dedham, MA, USA, #SIG-39320, 1:1000 for 1 h at room temperature) directed against the human amyloid peptide (amino acids 1–16), visualized by a fluorescent secondary antibody (Cy3, Jackson ImmunoResearch Laboratories Inc., West Grove, PA, USA) and thioflavin S (Sigma, 0.5% solution) staining against β-sheet structures in a double incubation. Additionally to standard protocol steps (drying, washing, primary and secondary incubations), slices were blocked with M.O.M blocking kit (Vector Laboratories Inc., Burlingame, CA, USA) before primary incubation to minimize unspecific secondary binding.

##### Imaging

Mosaic images including the whole cortex and hippocampus were recorded on a Zeiss AxioImager.Z1 microscope using a high aperture lens and an AxioVision 4.8 software driven AxioCam MRm (10× lens NA 0.45, 1× optocoupler, Zeiss, Jena, Germany).

##### Evaluation of Amyloid Depositions and Plaque Load

For determination of histopathological variables a uniform systematic random set of five sagittally cut 10 µm thick sections (deriving from the five different sagittal levels 2, 4, 7, 9, and 12) per brain were labelled and whole slice recordings were evaluated to collect a representative individual mean. Region size was measured by manual delineation of the hippocampus or neocortex. Labelings were quantified using automated image analysis software in a rater independent quantification (Image Pro Plus, version 6.2). IR objects were detected above an adaptive intensity threshold based on 8-bit 256 grey levels, which is defined as “mean signal in area of interest (AOI) +1.2× standard deviation of mean signal in AOI”. The level varied for the different labelings adjusted to the background fluorescence in the channel. Furthermore detected objects had to overcome a minimal size of 7 µm^2^. All variable values were transported automatedly into a “csv” format raw data file together with the image title and included object number, surface area, mean and sum signal as well as individual threshold levels. Values were normalized to the invidual AOI size for each section and region, the values of the five sections averaged to the one individual mean.

##### Statistical Analysis

Descriptive statistical analysis was performed on all evaluated parameters, Kolmogorov Smirnov tests were performed to test normal distribution of data. Data in graphs are represented as mean ± SEM. Outliers were detected by Grubbs’ test and were excluded from statistical analyses. Values of the five single measurements (from five different levels) per animal were averaged to one value per animal (individual mean) and group means were calculated with these averaged values. Group differences were calculated by an unpaired, two-tailed T-test.

### 3.4. Hepatotoxicity Studies in HepG2 Cells

In brief, HepG2 cells were cultured in 96-well plates at a density of 25,000 cells per well at 37 °C and 5% CO_2_ in growth medium (MEM, 10% FBS, 1% Penc/Strep, 2 mM glutamine, 1 mM sodium pyruvate). After 24 h, AVCRI104P4 and tacrine at the indicated concentrations were applied to the cells for a total of 24 h. Cell viability and cellular toxicity were determined according to MTT and LDH assays, respectively. Cell viability was determined by the MTT assay using a plate-reader (570 nm). MTT solution was added to each well in a final concentration of 0.5 mg/mL. After 2 h the MTT containing medium was aspired. Cells were lysed in 3% SDS and the formazan crystals were dissolved in i-PrOH/HCl. Optical density was measured with a plate-reader at wavelength 570 nm. Cell survival rate is expressed as optical density (OD). Data are shown as percentage of vehicle control.

Cellular toxicity was determined by the cytotoxicity detection kit (LDH, Roche, Mannheim, Germany). The assay was performed according to the manufacturer’s instructions. Optical density was measured with a plate-reader at wavelength 492 nm. Data are shown as percentage of vehicle control.

## 4. Conclusions

In a program directed to the development of disease-modifying anti-Alzheimer compounds, AVCRI104P4 recently emerged as a very promising drug candidate by virtue of its *in vitro* multitarget profile, which encompasses a very potent AChE inhibitory activity (low nanomolar range) and moderately potent inhibitory activities against BChE, Aβ aggregation, and BACE-1 (submicromolar or low micromolar range), as well as brain permeability. Because the potencies of a multitarget compound at its different targets may not necessarily have to be in a narrow range, *in vivo* efficacy studies may help to assess the adequate ratio of *in vitro* activities [19]. In this light, we embarked upon the *in vivo* testing of AVCRI104P4 to ascertain whether the anticholinesterase and anti-amyloid activities found *in vitro* might arise also *in vivo*.

In this work, we have scaled up all the synthetic steps leading to multigram amounts of AVCRI104P4, necessary to perform the planned *in vivo* studies. To this end, we have developed a novel methodology for the multigram chromatographic resolution of the immediate synthetic precursor huprine Y, which affords the required (–)-(7*S*,11*S*)-enantiomer with higher efficiency and in higher enantiomeric excess than the low scale procedure, previously reported by us [26].

*In vivo* studies have been conducted firstly in two transgenic *C. elegans* strains expressing Aβ3–42 or Aβ1–42 either constitutively (CL2006) or upon induction by a temperature shift (CL4176). Interestingly, AVCRI104P4 protected in a dose-dependent manner CL4176 nematodes against the paralysis caused by Aβ oligomers (IC_50_ 34 μM) and also counteracted the Aβ-induced toxicity in CL2006 worms, albeit to a lower extent. These effects are specific and not related to its AChE inhibitory activity as indicated by the absence of any protective effect observed with the AChE inhibitor aldicarb. A putative effect on Aβ production by BACE-1 inhibition or on Aβ aggregation can be also excluded because, on the one hand, CL2006 and CL4176 transgenic worms overexpress Aβ without intervention of BACE-1, and, on the other hand, AVCRI104P4 was not able to reduce insoluble Aβ deposits in CL2006 worms.

To gain further insight into the potential *in vivo* effects of AVCRI104P4 on amyloid pathology and cognition, behavioral, biochemical and histological studies were carried out in a well-established mouse model of AD, namely in APP_SL_ transgenic mice. AVCRI104P4, administered orally at a dose of 40 mg/kg/day for 3 months, led to a significant improvement of the short-term memory in the MWM compared with vehicle treated animals on the first training day, in spite of a severe muscular weakness displayed by some AVCRI104P4 treated animals. To overcome the problems due to that physical handicap, a one-week washout period without any treatment was applied before performing again the MWM. Unfortunately, after the washout period, transgenic mice that had received AVCRI104P4 did not display improved spatial learning and memory capabilities relative to transgenic controls. The observation of a clear cognitive effect while animals were being treated with AVCRI104P4 but the absence of positive effects one week after interruption of AVCRI104P4 treatment suggests that the potent AChE inhibitory activity that was found *in vitro* for AVCRI104P4 translates into an efficient compensation of the central cholinergic deficit and, hence, into a symptomatic relief, *i.e.*, that AVCRI104P4 exerts a purely symptomatic action. A disease-modifying effect by AVCRI104P4 would have led to a minor degree of neurodegeneration in treated mice and to a better performance in the MWM even after the washout period. The absence of a disease-modifying effect was also evident in the biochemical and histological studies, where AVCRI104P4 treated animals had similar levels of Aβ peptides in CSF and brain homogenates, as well as similar amyloid burden in cortex and hippocampus. These results suggest that AVCRI104P4 does not interfere either with Aβ production or aggregation.

In summary, AVCRI104P4 exerts a significant cholinergic cognitive effect in a transgenic mouse model of AD but it has no effect on Aβ production (Aβ peptides levels) and aggregation (amyloid burden) either in APP_SL_ mice or in *C. elegans*, contrary to our expectactions in the light of its *in vitro* multitarget profile including inhibition of BACE-1 and Aβ aggregation among other actions. Even though the mechanisms that are behind the protective effect of AVCRI104P4 in *C. elegans* remain unclear, observation of only a cholinergic but not an anti-amyloid effect for this compound in transgenic mice indicates that, besides a decreased toxicity, an improvement of the anti-amyloid/anticholinesterase activity ratio will be necessary in further lead optimization endeavours aimed at deriving a drug candidate with potential to modify the natural course of AD.

## Supplementary Materials

Supplementary materials can be accessed at: http://www.mdpi.com/1420-3049/20/03/4492/s1.
