# Supplementary material for: Multigram Synthesis and in Vivo Efficacy Studies of a Novel Multitarget Anti-Alzheimer’s Compound
Source: Molecules. 2015 Mar 10;20(3):4492–515. doi: 10.3390/molecules20034492 (PMC6272704; doi:10.3390/molecules20034492)
Supplement: Supplementary file 1 [file molecules-20-04492-s001.pdf]

Supplementary Materials

1. Synthesis of Racemic Huprine Y, (±)-1

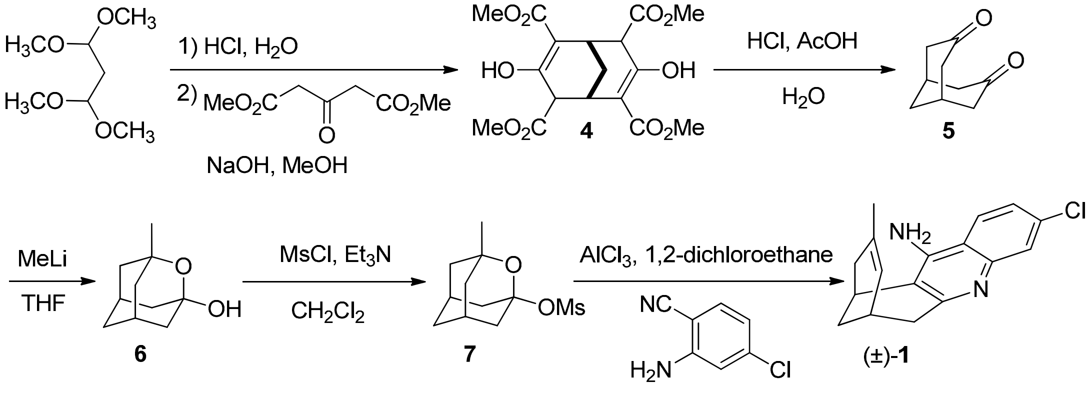

Scheme S1. Synthesis of intermediate racemic huprine Y, (±)-**1**.

1.1. Bicyclo[3.3.1]nonane-3,7-dione

To 1,1,3,3-tetramethoxypropane (226 mL, 225 g, 1.37 mol), 2 N HCl (690 mL, 1.38 mol) was added and the mixture was stirred at r.t. for 1.5 h, then alkalinized dropwise with 5 N NaOH (480 mL, 2.40 mol), and diluted with MeOH (670 mL). The resulting solution was treated with dimethyl 1,3-acetonedicarboxylate (387 mL, 459 g, 2.63 mol) and MeOH (460 mL). The reaction mixture was stirred at r.t. for 72 h and treated with concd. HCl (42 mL). The solid precipitate was separated by filtration *in vacuo*, washed with H_2O (2 × 50 mL) and dried under reduced pressure to give tetramethyl 3,7-dihydroxybicyclo[3.3.1]nona-2,6-diene-2,4,6,8-tetracarboxylate, **4**, as a yellowish solid (300 g, 59% yield), which was directly used in the next step without further purification.

A suspension of tetraester **4** (300 g, 781 mmol) in H_2O (650 mL) was treated with HOAc (1.31 L) and concd. HCl (650 mL). The reaction mixture was stirred under reflux for 24 h, allowed to cool to r.t. and extracted with CH_2Cl_2 (4 × 500 mL). The combined organic extracts were washed with 5 N NaOH (3 × 400 mL), dried over anhyd. Na_2SO_4, and evaporated under reduced pressure to give diketone **5** as a beige solid (60.0 g, 51% yield). The IR, ^{1}H- and ^{13}C-NMR data of **5** coincided with those reported [1].

1.2. 3-Methyl-2-oxa-1-adamantanol

A Et_2O solution of MeLi (1.6 M, 381 mL, 610 mmol) was cooled to 0 °C and treated dropwise with a solution of diketone **5** (62.0 g, 408 mmol) in anhydrous THF (1.3 L). The reaction mixture was stirred at 0 °C for 35 min, treated dropwise with sat. aq. NH_4Cl (950 mL), and extracted with Et_2O (3 × 760 mL). The combined organic extracts were dried over anhyd. Na_2SO_4, and evaporated under reduced pressure to give oxaadamantanol **6** (61.3 g, 89% yield) as a yellow solid, whose IR, ^{1}H- and ^{13}C-NMR data coincided with those previously reported [2].

1.3. 3-Methyl-2-oxa-1-adamantyl Methanesulfonate

A solution of oxaadamantanol **6** (59.5 g, 354 mmol) and anhyd. Et_3N (70.9 mL, 51.5 g, 509 mmol) in anhyd. CH_2Cl_2 (823 mL) was cooled to −10 °C and treated dropwise with MsCl (43.8 mL, 64.8 g, 566 mmol). The reaction mixture was stirred at −10 °C for 30 min and poured onto a mixture of 10% aq. HCl (300 mL) and crushed ice (140 mL). The phases were separated and the aqueous layer was extracted with CH_2Cl_2 (2 × 350 mL). The combined organic extracts were washed with sat. aq. $NaHCO_3$ (420 mL) and brine (300 mL), dried over anhyd. Na_2SO_4, and concentrated under reduced pressure to give mesylate **7** (86.5 g, 99% yield) as a yellowish solid, whose IR, ^1H- and ^{13}C-NMR data coincided with those previously reported [2].

1.4. (±)-12-Amino-3-chloro-6,7,10,11-tetrahydro-9-methyl-7,11-methanocycloocta[b]quinoline, (±)-Huprine Y

To $AlCl_3$ (56.0 g, 420 mmol), a solution of mesylate **7** (85.0 g, 346 mmol) in anhyd. 1,2-dichloroethane (500 mL) was added dropwise. The resulting mixture was stirred under reflux for 30 min, treated dropwise with a suspension of 2-amino-4-chlorobenzonitrile (58.0 g, 380 mmol) in anhyd. 1,2-dichloroethane (1 L). The reaction mixture was stirred under reflux overnight, allowed to cool to r.t., diluted with H_2O (800 mL) and THF (600 mL), alkalinized with 5 N NaOH (600 mL) and stirred at r.t. for 30 min. The phases were separated and the aqueous layer was extracted with EtOAc (6 × 300 mL). The combined organic extracts were dried over anhyd. Na_2SO_4, filtered and concentrated under reduced pressure to give a solid (104 g), which consisted of a mixture of starting 2-amino-4-chlorobenzonitrile/(±)-**1** in a 1:1 approximate ratio (^1H-NMR). This mixture was dissolved in CH_2Cl_2/MeOH 5:3 (800 mL), treated with a MeOH solution of HCl (1.4 N, 116 mL) and evaporated under reduced pressure. After washing of the resulting solid (110 g) with refluxing EtOAc (1.1 L + 0.5 L), (±)-huprine Y·HCl, (±)-**1**·HCl (41.0 g, 37% yield) was separated by filtration *in vacuo*. The IR, ^1H- and ^{13}C-NMR data of (±)-**1**·HCl coincided with those previously reported [3].

2. Synthesis of Chloroderivative 2

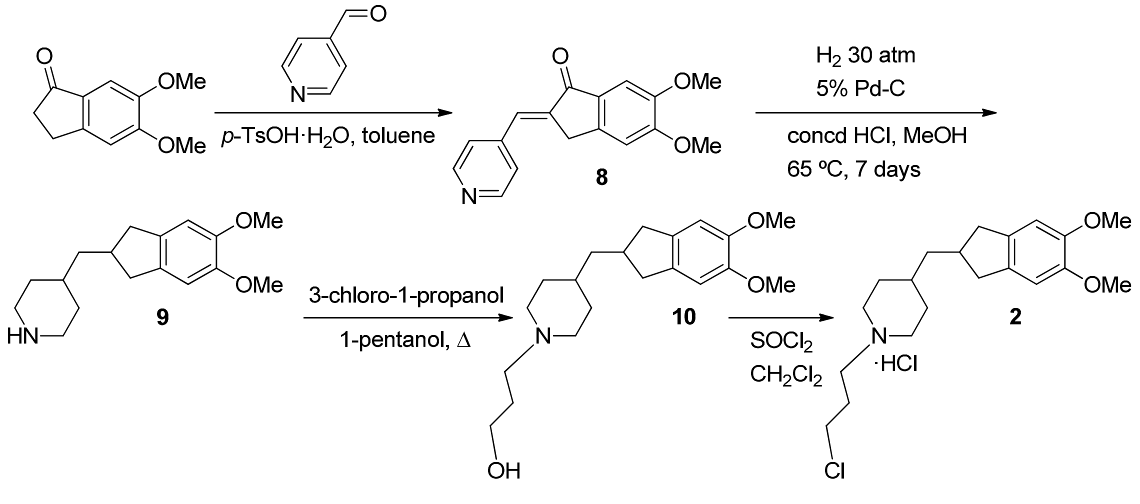

Scheme S2. Synthesis of intermediate chloroderivative **2**.

2.1. (E)-5,6-Dimethoxy-2-[(4-pyridyl)methylene]-1-indanone

A solution of 5,6-dimethoxy-1-indanone (20.0 g, 104 mmol) in toluene (250 mL) was treated with pyridine-4-carboxaldehyde (13.7 mL, 15.4 g, 144 mmol) and *p*-TsOH·H_2O (30.4 g, 160 mmol). The reaction mixture was heated under reflux for 6 h, allowed to cool to r.t. and filtered. The solid was washed with 10% aq. $NaHCO_3$ (400 mL) and H_2O (400 mL) and dried at 50 °C/20 Torr to give **8** (28.5 g, 98% yield) as a yellow solid, whose IR, ^{1}H- and ^{13}C-NMR data coincided with those previously reported [4].

2.2. 5,6-Dimethoxy-2-[(4-piperidinyl)methyl]indane

A suspension of **8** (28.5 g, 101 mmol), concd. HCl (17 mL) and 5% Pd/C (containing 50% of H_2O, 19.0 g) in MeOH (480 mL) was hydrogenated at 30 atm and 65 °C for 7 days. The resulting suspension was filtered and evaporated under reduced pressure. The residue was taken in 5 N NaOH (250 mL) and extracted with CH_2Cl_2 (2 × 250 mL) and EtOAc (2 × 250 mL). The combined organic extracts were dried over anhyd. Na_2SO_4 and evaporated under reduced pressure to give piperidine **9** (26.9 g, 97% yield) as a beige solid, whose IR, ^{1}H- and ^{13}C-NMR data coincided with those previously reported [4].

2.3. 2-{[1-(3-Hydroxypropyl)piperidin-4-yl]methyl}-5,6-dimethoxyindane

A solution of piperidine **9** (51.0 g, 185 mmol) and 3-chloro-1-propanol (30.9 mL, 34.9 g, 370 mmol) in 1-pentanol (700 mL) was stirred under reflux for 48 h. The resulting suspension was allowed to cool to r.t., diluted with EtOAc (1 L) and extracted with 1 N HCl (3 × 300 mL). The combined aqueous extracts were washed with EtOAc (4 × 350 mL), alkalinized with NaOH pellets to pH 14, and extracted with CH_2Cl_2 (4 × 450 mL). The combined organic extracts were dried over anhyd. Na_2SO_4 and evaporated under reduced pressure to give a grey solid that was purified through column chromatography (40–60 µm silica gel, CH_2Cl_2/MeOH/50% aq. NH_4OH mixtures, gradient elution). On elution with CH_2Cl_2/MeOH/50% aq. NH_4OH 92:8:0.5, alcohol **10** (12.1 g, 20% yield) was isolated. On elution with CH_2Cl_2/MeOH/50% aq. NH_4OH 80:20:0.5, starting piperidine **9** (9.18 g) was recovered unchanged.

Evaporation of the initial EtOAc phase gave a brown oil that was purified through column chromatography (40–60 µm silica gel, CH_2Cl_2/MeOH/50% aq. NH_4OH mixtures, gradient elution). On elution with CH_2Cl_2/MeOH/50% aq. NH_4OH 92:8:0.5, an additional amount of alcohol **10** (16.5 g, 27% yield, 47% total yield) was isolated. The IR, ^{1}H- and ^{13}C-NMR data of **10** coincided with those previously reported [5].

2.4. 2-{[1-(3-Chloropropyl)piperidin-4-yl]methyl}-5,6-dimethoxyindane

A solution of alcohol **10** (4.70 g, 14.1 mmol) in anhyd. CH_2Cl_2 (90 mL) was cooled to 5 °C and treated dropwise with $SOCl_2$ (54.4 mL, 88.7 g, 746 mmol). The reaction mixture was stirred under reflux for 4 h, allowed to cool to r.t. and evaporated under reduced pressure. The resulting brown oil was repeatedly taken in CH_2Cl_2 (5 × 70 mL) and evaporated under reduced pressure to remove residual $SOCl_2$. A beige solid (6.50 g), which seemed to consist mainly of the desired chloroderivative **2** (^{1}H-NMR), was obtained and directly used in the next step without further purification.

Table S1. Measurement parameters for the evaluation of amyloid depositions and plaque load.

Thresholds	Minimum	Dynamic Factor	Minimum Object Size
**6E10 amyloid plaques**	25	1.8	>7 µm^2
**ThioS plaques**	25	1.6	>7 µm^2

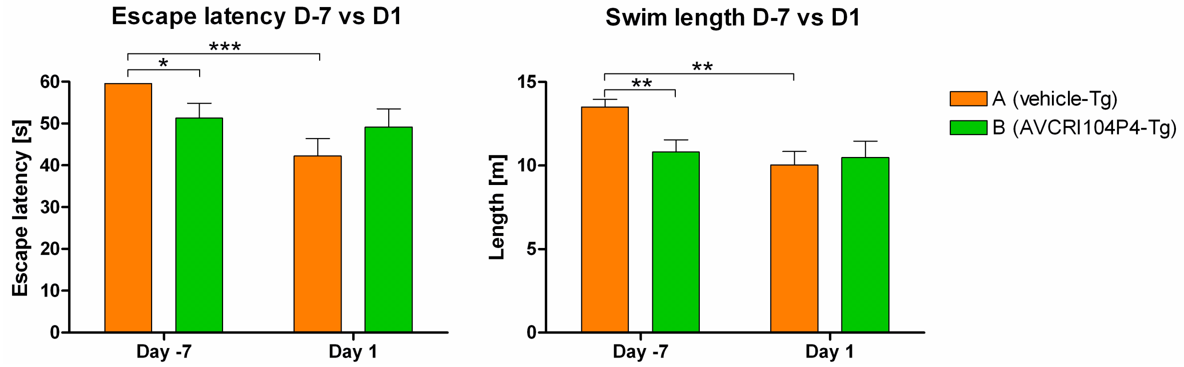

Figure S1. Comparison of escape latency and swim length on training days -7 and 1 (MWM). Graphs represent the escape latency (left graph) as well as length of swimming path (right graph) of the MWM of hAPP$_{SL}$ transgenic animals treated with vehicle only (group A, orange) or with AVCRI104P4 (group B, green) for 3 months. Data are presented as mean ± SEM for each treatment group and day. Statistical significant differences were detected in vehicle treated animals on day -7 compared to day 1 in terms of escape latency and swim length using unpaired *t*-test. * indicates $p < 0.05$, ** $p < 0.01$, *** $p < 0.001$.

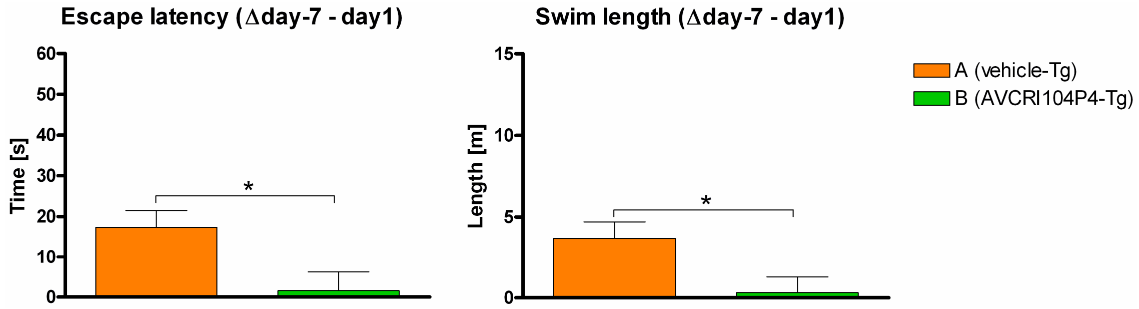

Figure S2. Escape latency and swim length Δ days -7 to day 1 (MWM). Graphs represent the difference of day -7 and day 1 in time (left graph) and length (right graph) to reach the platform position in the MWM. APP$_{SL}$ transgenic animals were treated with vehicle only (group A, orange) or with AVCRI104P4 (group B, green) for 3 months. Data are presented as mean ± SEM for each treatment group. Statistical significant differences were detected in vehicle treated animals (group A) compared to group B in terms of escape latency and swim length using unpaired *t*-test: * indicates $p < 0.05$.

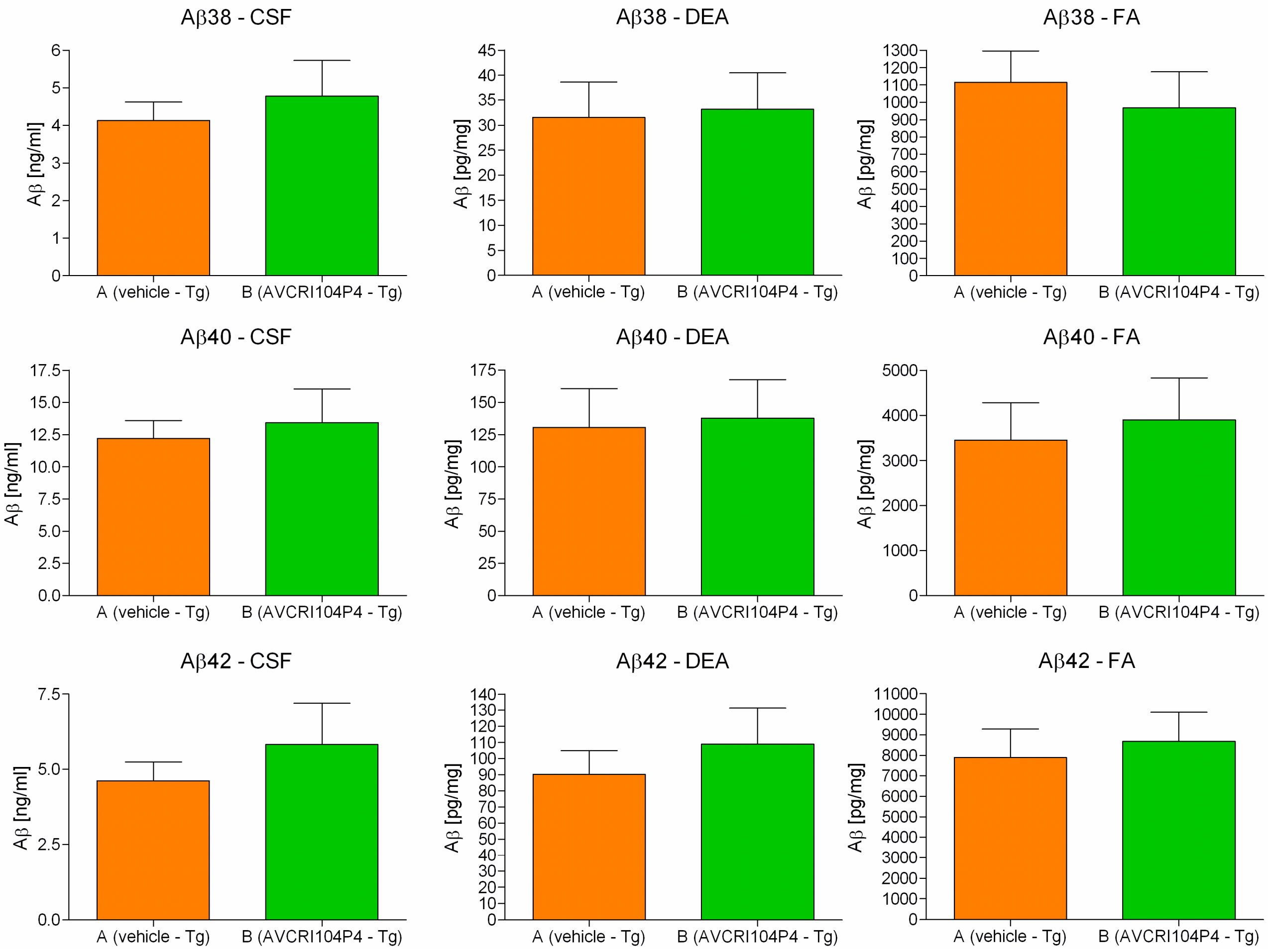

Figure S3. Effects of treatment with AVCRI104P4 on Aβ levels in CSF and brain homogenates. Graphs represent Aβ38 (upper line), Aβ40 (middle line), and Aβ42 (lower line) in CSF (left column), DEA brain homogenate preparation (middle column), and FA brain homogenate preparation (right column). Data are presented as group mean + SEM. No statistical significant group differences were detected.

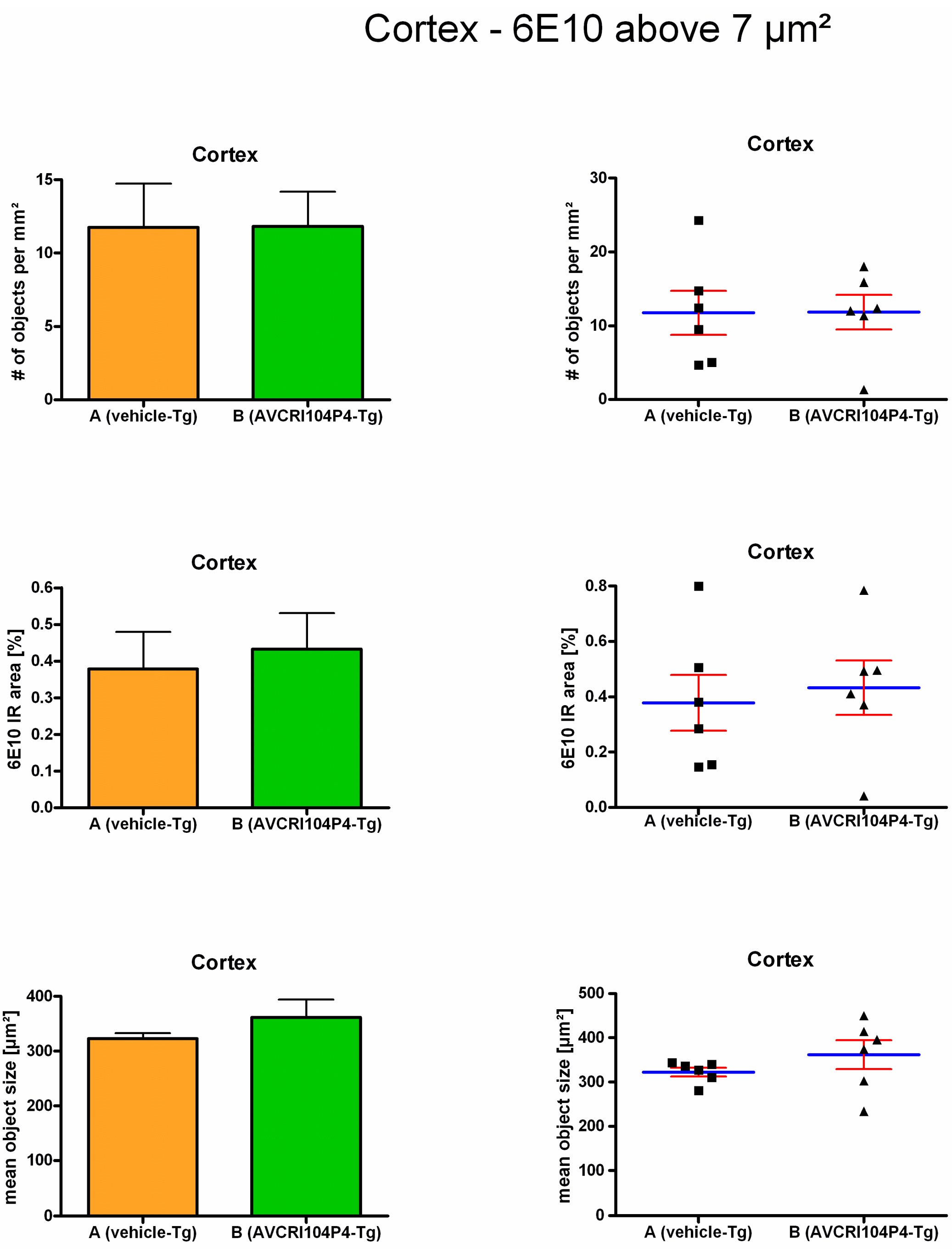

(A)

Figure S4. *Cont.*

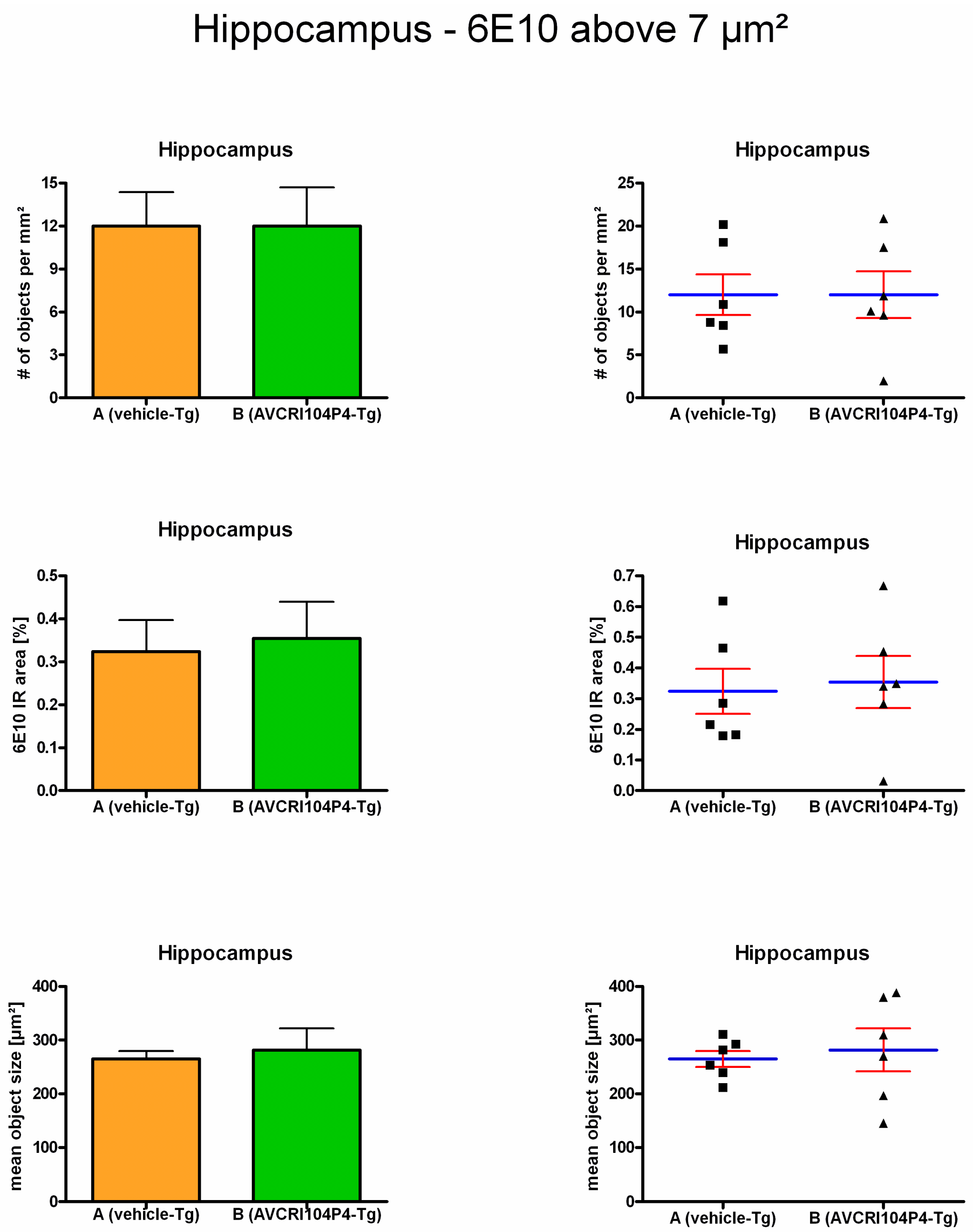

(B)

Figure S4. Effects of treatment with AVCRI104P4 on amyloid load (6E10 IHC) (**A**) in the cortex and (**B**) in the hippocampus. Graphs represent the number of 6E10 immunoreactive (IR) objects, 6E10 IR area and mean size of 6E10 IR objects in the cortex or hippocampus of transgenic mice treated with vehicle (group A, orange) or AVCRI104P4 (group B, green). No statistical significant differences were detectable among the treatment groups. Data are represented as mean + SEM or scatter ± SEM.

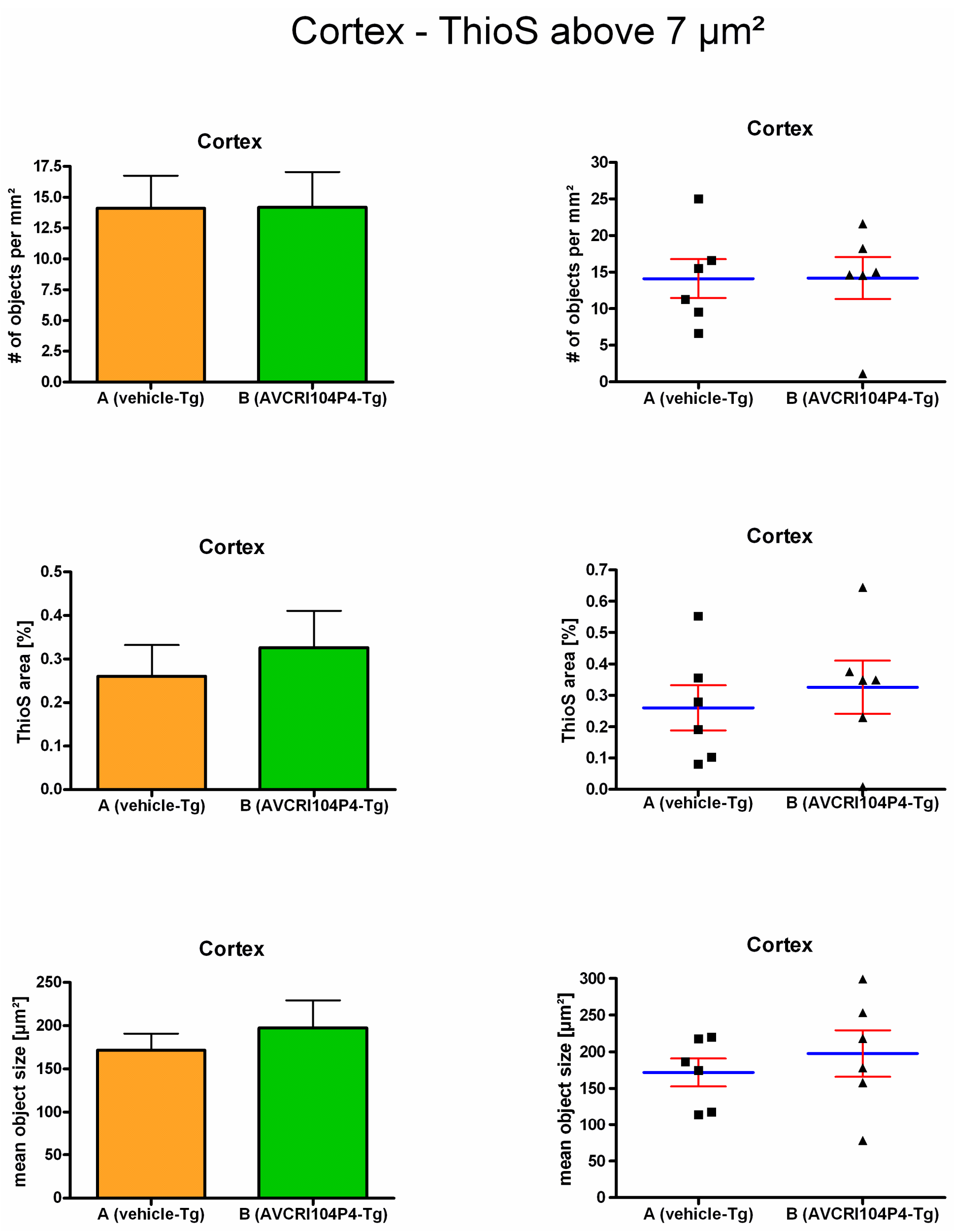

(A)

Figure S5. *Cont.*

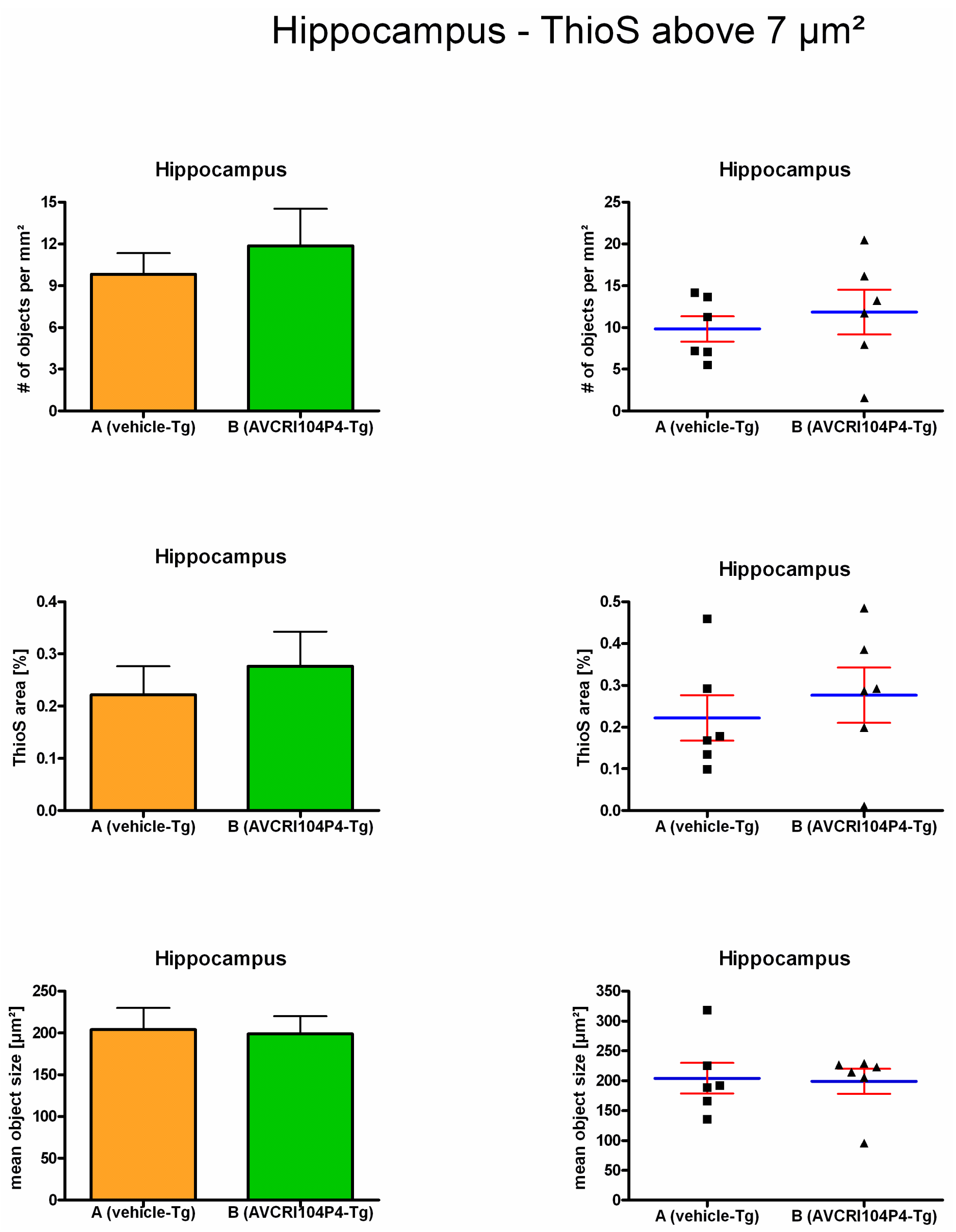

(**B**)

Figure S5. Effects of treatment with AVCRI104P4 on β-sheeted plaque cores (ThioS staining) (**A**) in the cortex and (**B**) in the hippocampus. Graphs represent the number of ThioS objects, ThioS area and mean size of ThioS objects in the cortex or hippocampus of transgenic mice treated with vehicle (group A, orange) or AVCRI104P4 (group B, green). No statistical significant differences were detectable among the treatment groups. Data are represented as mean + SEM or scatter ± SEM.

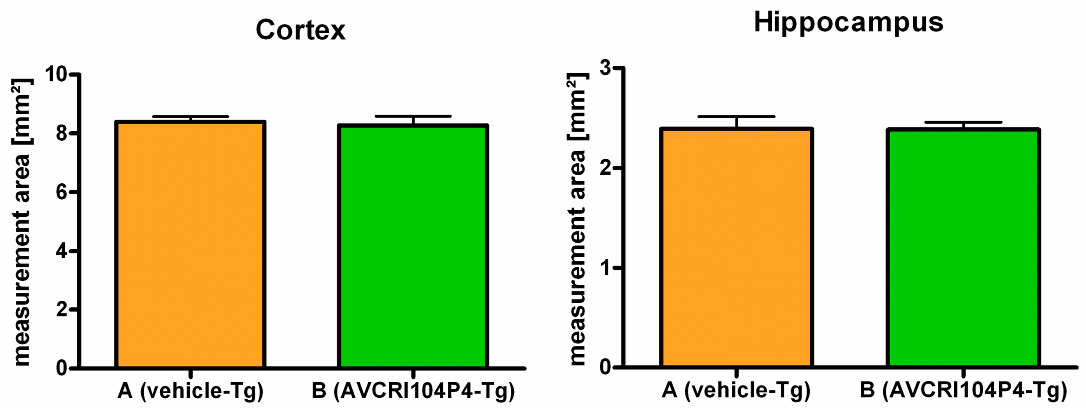

Figure S6. Effects of treatment with AVCRI104P4 on region area of 6E10/ThioS labeling. Graphs represent the region areas of the cortex and the hippocampus of transgenic mice treated with vehicle (group **A**, orange) or AVCRI104P4 (group **B**, green). No differences were detectable excluding problems in the cutting and IHC procedures and to a certain degree a treatment or genotype induced atrophy. Data are represented as mean + SEM.

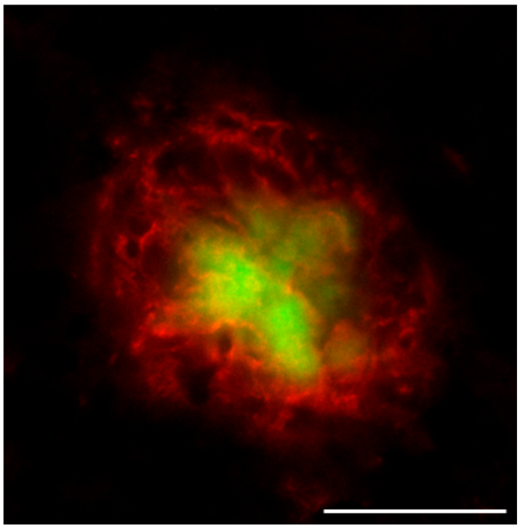

Figure S7. Exemplar high magnified plaque visualized by 6E10 and ThioS labeling in the cortex of an APP_{SL} mouse. 6E10 IHC (red) and thioflavin S staining (green). Image shows the principal construction of a mature, neuritic plaque with a dense thioflavin S positive β-sheet core with surrounding and partly overlapping areas between β-sheets and diffuse 6E10 IR human amyloid in the surrounding of the plaque. Cutout from 40×, 2D collapsed image from 3D Z-stacks, scale bar 30 μm.

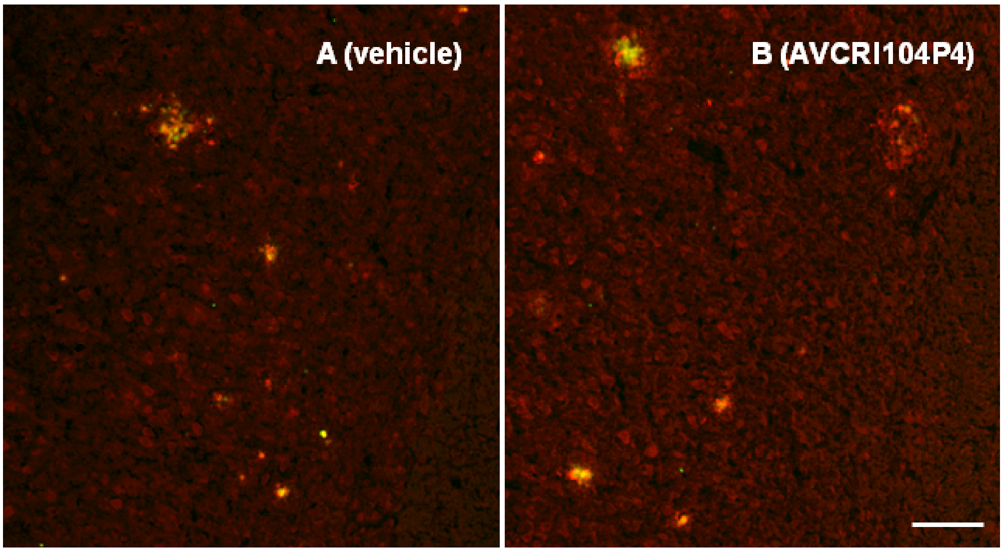

Figure S8. Representative cutouts from images used for quantification showing the primary somatosensory cortex of APP_{SL} mice. 6E10 IHC (red) and thioflavin S staining (green) in the cortex of APP_{SL} transgenic mice treated with vehicle (group **A**) and AVCRI104P4 (group **B**). No obvious morphological differences of plaques were detectable after treatment with AVCRI104P4, also overall intracellular somal amyloid labeling stayed comparable. Scale bar 50 μm.

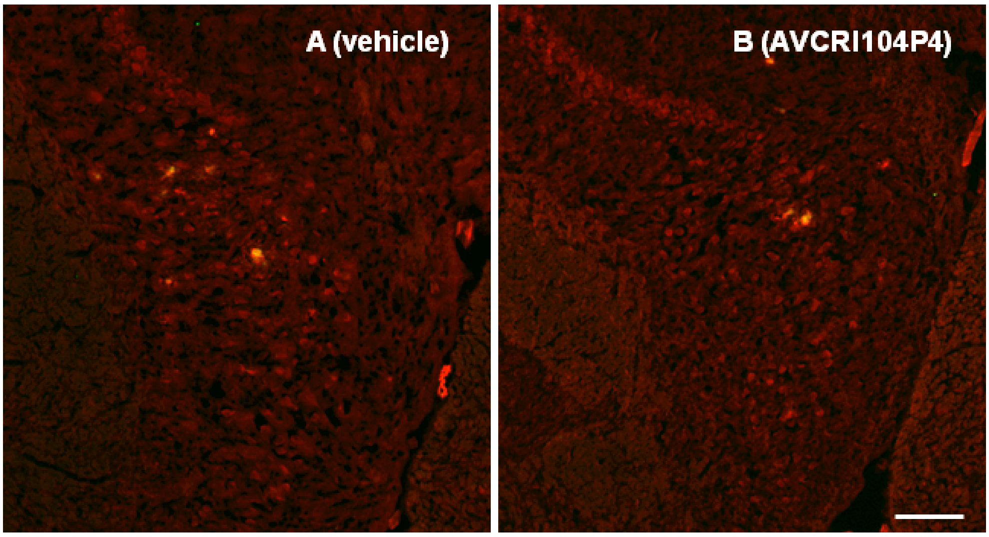

Figure S9. Representative cutouts from images used for quantification showing the hippocampal subiculum of APP_{SL} mice. 6E10 IHC (red) and thioflavin S staining (green) in the hippocampal subiculum of transgenic mice treated with vehicle (group **A**) and AVCRI104P4 (group **B**). No obvious morphological differences of plaques were detectable after treatment with AVCRI104P4, also overall intracellular somal amyloid labeling stayed comparable. Scale bar 50 μm.

References

1. Bertz, S.H. Tetramethyl 3,7-dihydroxybicyclo[3.3.1]nona-2,6-diene-2,4,6,8-tetracarboxylate: A useful companion to Meerwein's ester. Topological analysis of bicyclo[3.3.1]nonane synthesis. *J. Org. Chem.* **1985**, *50*, 3585–3592.
2. Camps, P.; El Achab, R.; Font-Bardia, M.; Görbig, D.; Morral, J.; Muñoz-Torrero, D.; Solans, X.; Simon, M. Easy synthesis of 7-alkylbicyclo[3.3.1]non-6-en-3-ones by silica gel-promoted fragmentation of 3-alkyl-2-oxaadamant-1-yl mesylates. *Tetrahedron* **1996**, *52*, 5867–5880.
3. Camps, P.; Contreras, J.; Font-Bardia, M.; Morral, J.; Muñoz-Torrero, D.; Solans, X. Enantioselective synthesis of tacrine-huperzine A hybrids. Preparative chiral MPLC separation of their racemic mixtures and absolute configuration assignments by X-ray diffraction analysis. *Tetrahedron Asymmetry* **1998**, *9*, 835–849.
4. Camps, P.; Formosa, X.; Galdeano, C.; Gómez, T.; Muñoz-Torrero, D.; Scarpellini, M.; Viayna, E.; Badia, A.; Clos, M.V.; Camins, A.; *et al.* Novel donepezil-based inhibitors of acetyl- and butyrylcholinesterase and acetylcholinesterase-induced β-amyloid aggregation. *J. Med. Chem.* **2008**, *51*, 3588–3598.
5. Viayna, E.; Gómez, T.; Galdeano, C.; Ramírez, L.; Ratia, M.; Badia, A.; Clos, M.V.; Verdaguer, E.; Junyent, F.; Camins, A.; *et al.* Novel huprine derivatives with inhibitory activity toward β-amyloid aggregation and formation as disease-modifying anti-Alzheimer drug candidates. *ChemMedChem* **2010**, *5*, 1855–1870.
